# Recovery of Polyhydroxyalkanoates From Single and Mixed Microbial Cultures: A Review

**DOI:** 10.3389/fbioe.2021.624021

**Published:** 2021-02-10

**Authors:** Giorgia Pagliano, Paola Galletti, Chiara Samorì, Agnese Zaghini, Cristian Torri

**Affiliations:** ^1^Department of Chemistry “Giacomo Ciamician”, University of Bologna, Ravenna, Italy; ^2^CIRI-Fonti Rinnovabili, Ambiente, Mare ed Energia, Ravenna, Italy

**Keywords:** polyhydroxyalkanoates, extraction methods, green solvents, mixed microbial cultures, single microbial strains, surfactants, cell lysis

## Abstract

An overview of the main polyhydroxyalkanoates (PHA) recovery methods is here reported, by considering the kind of PHA-producing bacteria (single bacterial strains or mixed microbial cultures) and the chemico-physical characteristics of the extracted polymer (molecular weight and polydispersity index). Several recovery approaches are presented and categorized in two main strategies: PHA recovery with solvents (halogenated solvents, alkanes, alcohols, esters, carbonates and ketones) and PHA recovery by cellular lysis (with oxidants, acid and alkaline compounds, surfactants and enzymes). Comparative evaluations based on the recovery, purity and molecular weight of the recovered polymers as well as on the potential sustainability of the different approaches are here presented.

## Introduction

Polyhydroxyalkanoates are a family of bio-polyesters constituted by 3-hydroxy acid monomers (e.g., mainly 3-hydroxybutyric acid copolymerized with longer monomers as 3-hydroxyvaleric acid) and produced by bacterial fermentation as intracellular carbon and energy storage. Despite their potential in the scenario of fossil plastic replacement due to thermo-physical properties like petrochemically-derived plastics, PHAs are currently more costly than standard petrochemical plastics (1.18–6.12 €/kg *vs.*<1 €/kg, Saavedra del Oso et al., 2020) and cover niche-market high-value applications (Samorì et al., 2015b; Valentino et al., 2017). The combination of two factors determines the current PHA high production cost: upstream costs and downstream costs. A huge effort has been devoted at reducing the upstream costs by investigating both the suitability of cheap raw materials as feed for PHA-producing bacteria and the suitability of alternative microbial consortia for fermenting unusual feedstock; to this purpose different kinds of wastewater streams (food waste, sugar cane molasses, olive mill wastewater, waste activated sludge, paper mill wastewater, cheese whey, Mannina et al., 2019) have been used for feeding mixed microbial cultures (MMC) that proved to have wider metabolic potential than single strains and to be cheaper in terms of operative costs (Dias et al., 2006; Serafim et al., 2008a; Mannina et al., 2020).

On the other hand, downstream processes (PHA recovery and purification) are among the least investigated aspects of the whole PHA production chain but the most impacting ones in terms of economic weight (Jiang et al., 2015; Koller et al., 2017; Saavedra del Oso et al., 2020). High energy consumption is intrinsic in the overall PHA production life cycle, especially during the PHA downstream processing, and this aspect is clearly highlighted by life cycle assessment (LCA) studies that compare PHA and fossil plastics, having the firsts a higher carbon footprint despite being biobased and biodegradable (Saavedra del Oso et al., 2020).

The use of (i) appropriate organic solvents to extract PHA granules from inside the bacterial cells, or (ii) additives/chemical agents to disrupt cellular matrix (dissolution of non-PHA cell mass, NPCM) and releasing intracellular PHA, are the two approaches of choice in the downstream phase, often coupled with pretreatments (e.g., with an oxidant, or thermally assisted) to enhance the permeability of cellular membranes. Both are characterized by peculiar advantages and undeniable drawbacks (Samorì et al., 2015b): PHA-friendly solvents, especially the chlorinated ones, provide high extraction yields and high polymer quality (high molecular weight and low impurities) but they are often hazardous for the environment and for the operators. Moreover, the solvent-based processes require relevant operational costs due to the high quantity of solvents that has to be used (up to 20-folds the PHA-rich biomass) and the high amount of energy employed for solvent evaporation and partial/total water removal from bacterial biomass to improve the contact between hydrophobic solvents and PHA granules stored inside the cells (Koller et al., 2013; Madkour et al., 2013). The additives for achieving cellular lysis (enzymes, surfactants, oxidants, alkali) can be directly applied to microbial cultures, by-passing the drying of the biomass, but they can affect the characteristics of the extracted polymer, leading to PHA degradation and reduction of molecular weight (Kosseva and Rusbandi, 2018; Mannina et al., 2020). Moreover, these agents are often non-recyclable and consequently, the remaining aqueous solutions are wasted and must be treated.

The search for more sustainable alternatives for PHA extraction that could decrease both the environmental and economic impact of the current approaches has been investigated in the last years: linear and cyclic carbonates (Fiorese et al., 2009; Samorì et al., 2015a, b), ethyl acetate (Riedel et al., 2013), methyl isobutyl ketone (Riedel et al., 2013), ionic liquids, and supercritical fluids (Hampson and Ashby, 1999) are some examples of solvents used in the first approach, whereas recyclable surfactants are among the alternatives developed within the second approach (Samorì et al., 2015b; Mannina et al., 2019). In this framework it has also emerged that PHA-accumulating bacteria do not behave in the same way toward organic solvents or chemical additives: MMCs, cheaper in terms of upstream than single strains, seem more resistant to cell hydrolysis probably because of strong and complex extracellular biomass matrix that contains the PHA accumulating cells (Patel et al., 2009; Samorì et al., 2015b). This aspect poses a further complication in making the PHA-production process economically competitive with respect to fossil-based plastics and other bioplastics already on the market.

In this review, an overview of the various downstream approaches applied to single strains and MMCs for recovering PHAs is presented. These methods have been divided into two main categories: recovery with solvents and recovery by cellular lysis. A comparative assessment among them was shown by considering the recovery, purity, molecular weight, and polydispersity index of the recovered polymer. The last three properties, in fact, are among the most important parameters for determining the applicability of each specific PHA in the various fields; if PHA purity is strictly correlated to human-related applications (e.g., biomedical ones), PHA molecular weight, and polydispersity index are detrimental for PHA processability. In fact, mechanical properties of polymers (e.g., the tensile strength) are affected by their molar mass; molecular weight values above 0.5 MDa and polydispersity index below 3 are usually considered acceptable thresholds for these thermoplastic polymers, being typical of quite homogeneous chain lengths that can be processed through injection molding techniques without any compromising reduction in the total polymer length (Fiorese et al., 2009). Some studies have reported for example that a molecular weight below 0.1 MDa causes severe deterioration of the mechanical properties for P(HB-HV) (Burniol-Figols et al., 2020).

It is worth mentioning that PHAs are currently used as a chemically-extracted bulk material. However, it has been demonstrated that the extraction process, independently by being a solvent-based or a cellular lysis approach, has a crucial role in determining the properties of the extracted PHA, both in terms of crystallinity and purity, altering the original morphology of PHA granules. In fact, storage PHB (high molecular weight polymer with > 10^3^ 3-HB units) and related PHA are accumulated intracellularly in the form of granules whose surface is surrounded by a considerable number of proteins (about 1.9 wt%), much more than those essential for PHA synthesis (Jendrossek and Pfeiffer, 2014). These structural, biosynthetic, catabolic, and even regulatory proteins, embedded in lipid monolayer (Merrick and Doudoroff, 1964; Mayer and Hoppert, 1997), create a surface layer around the polymer core (Jendrossek and Pfeiffer, 2014), and this complex structure have suggested a wider function of PHB/PHA granules (thus named “carbonosomes,” Jendrossek, 2009) a part being an energy and carbon storage. When PHA granules are exposed to (bio)chemical (e.g., extraction with alkaline compounds, solvents, enzymes) or physical processes (freezing, pelleting by centrifugation), they rapidly undergo denaturation processes (Merrick and Doudoroff, 1964) and become more crystalline (typical degree of crystallinity 50–60%, Jendrossek, 2007) than the native PHB granules (in which the polymer chains are in an amorphous state due to a certain amount of water that acts as a plasticizer and prevents crystallization, Grage et al., 2009); moreover, the extracted PHA granules seem to retain the proteinaceous surface layer typical of the native PHB granules (Kuchta et al., 2007), meaning that a 100% purity of the granules is hard to be achieved. To avoid denaturation during the isolation process, it has been claimed that native PHA granules must be recovered by using mechanic (e.g., French Press) or enzymatic cell lysis followed by density gradient centrifugation; in this way, the particular spherical structure of native PHA granules and their shell-core composition could be maintained and thus exploitable in a broad range of applications in biotechnology and medicine, from protein purification to drug delivery (Grage et al., 2009). Although this approach could drastically increase the applicability of PHA in unexplored fields due to the extraction of such peculiar PHB-carbonosomes, this review will mainly focus on PHA as low-medium cost bulk material, potentially capable of playing a role in the future bioplastic scenario.

## PHA Extraction: Process Steps and Issues

### Methodologies to Determine the PHB/PHA Content Inside Microbial Cells, Recovery, Purity, Molecular Weight and Polydispersity Index

This review aims at comparing various extraction approaches by considering four different parameters: polymer recovery, purity, molecular weight, and polydispersity index of the recovered polymer. Therefore, their definitions and the most common methodologies to quantify all of them have been initially reported in this section.

#### PHA Recovery (%)

The recovery of PHA is directly correlated to PHA extraction yield, purity of the extracted PHA, and initial PHA content inside microbial cells as follows:

$$\left[\text{PHA}\text{recovery}\left(\%\right)=\frac{\text{PHA}\text{yield}{\left(\mathrm{wt}\%\right)}^{*}\mathrm{PHA}\mathrm{purity}\left(\%\right)}{\text{PHA}\,\text{amount}\,\text{in}\,\text{the}\,\text{microbial}\,\text{cells}\,\left(\mathrm{wt}\%\right)}\right]$$

#### PHA Yield (wt%)

The yield of the extracted PHA is usually calculated gravimetrically on a microbial biomass weight basis (wt%). The weight of the recovered polymer can be achieved after: (i) evaporating the solvent used for the extraction until reaching a constant PHA weight, (ii) adding an anti-solvent to the solvent used for the extraction (e.g., EtOH added to chlorinated compounds), (iii) lysing the microbial cells. If an anti-solvent or a cell-lysis approach is used, the PHA granules are recovered by centrifugation, washed with the anti-solvent or H_2_O, and then dried until reaching a constant weight.

#### PHA Purity (%)

The purity of the extracted PHA can be determined by various approaches that fall into two main categories:

(i) methods to quantify the impurities associated with the extracted polymer, typically oriented toward specific classes of bacterial contaminants like proteins, endotoxins, or lipids:
  • Proteins (about 1.9 wt%) are strictly associated to PHA granules, forming the so-called “carbonosomes” structure (Jendrossek, 2009) that is recalcitrant independently by the recovery approach (solvents or lytic agents); this residual content is directly quantifiable through the Lowry method (Lowry et al., 1951) or indirectly estimable through elemental analysis from the N amount of the sample (assuming that proteins have a mean nitrogen content of about 6%, Sosulski and Imafidon, 1990). A possible strategy to reduce protein residual content is the application of proteases after the extraction process.
  • Pyrogenic lipopolysaccharides (LPS) form part of the cell wall of Gram-negative bacteria and are released into the environment when the membrane of these bacteria is broken; LPS represent the so-called “endotoxins” and are associated to the extracted PHA independently by the recovery approach; however, it has been proved that solvents extraction (e.g., with chloroform) gives lower endotoxin level (three orders of magnitude) than cell-lysis (Lee et al., 1999). LPS residual content can be semi-quantitatively determined by *ad hoc* tests like the Limulus Amebocyte Lysate (LAL) test, whose principle is based on the gelation process that occurs from the coagulation of the proteins caused by the presence of endotoxins (Lee et al., 1999). Possible strategies to reduce LPS residual content are the application of alkaline or oxidizing post-treatments to the extracted PHA (however causing the hydrolysis of PHA itself), repeated polymer dissolution-precipitation cycles (however causing a massive solvents consumption), or filtration through charcoal (however causing a considerable loss of PHA, Wampfler et al., 2010a; Koller et al., 2013).
  • Lipids are usually found associated with extracted PHA if solvent approaches are used; this occurs because of the “like dissolves like” rule of thumb, according to which non-polar solvents used to solubilize PHA are also capable of dissolve non-polar solutes like lipids. Lipidic residual content is directly quantifiable through various techniques like thin-layer chromatography (TLC), gas chromatography (GC), liquid chromatography (LC), enzyme-linked immunosorbent assays (ELISA), nuclear magnetic resonance (NMR), and mass spectrometry (MS) (Li et al., 2014). Due to the similar chemical nature of lipids and PHA, post-treatment purification is quite hard; however, it can be useful to pretreat (degrease) the microbial biomass with solvents like methanol, ethanol, acetone, or supercritical CO_2_ (scCO_2_) that are scarcely suitable for PHA but not for lipids, weakening at the same time the cell envelops (Koller et al., 2013).
(ii) methods to quantify the PHA material in the mass recovered through solvent extraction or cell-lysis like methanolysis coupled to chromatographic analysis or thermogravimetric (TGA) analysis (see below). The main issue of the methods that calculate the purity level from the PHA content in the recovered material is that, even with a minor relative standard deviation (RSD) of the replicas, the absolute error becomes intrinsically comparable to the distance between measured purity and 100%. Gas chromatographic analysis, for example, typically brings 1–2% RSD due to imprecisions in weighing or dilution, thus more than 4–6 replicas (of a perfectly homogenous material) are needed to reduce the 95% confidence range of the data to 1%. If the confidence range is 1%, 99% purity can be investigated.

Given that pretreatment or derivatization procedures usually add a significant variability to the methods (RSD can exceed 5%) the number of replicas required to prove higher purities (>99%) becomes unfeasible. TGA analysis is characterized by higher precision (RSD < 1%), therefore it can be easily used (with a few replicas) to assess purity levels until 99%. Nonetheless, given the limitations highlighted above, all known methods that quantify the PHA material in the mass recovered cannot be used for a reliable determination of purities higher than 99%. When the interest is in determining a minimal amount of impurities, which is the case of higher purity investigation, direct quantification of impurities is markedly more efficient.

#### PHA Amount in Microbial Cells (wt%)

Several methods have been suggested in the last 50 years to quantify the PHA amount inside microbial cells and their monomeric composition (e.g., chromatographic, turbidimetric, spectrophotometric approaches) (Williamson and Wilkinson, 1958; Ward and Dawes, 1973; Braunegg et al., 1978; Gorenflo et al., 1999). Among them, methanolysis coupled to chromatographic analysis is considered one of the most reliable and accurate methods (Braunegg et al., 1978). It basically consists of a transesterification reaction (depolymerization) in methanol (>3 h at 100°C) catalyzed by H_2_SO_4_ to give the methylesters of PHA monomers (e.g., methyl 3-hydroxybutyrate from PHB), that are extracted by chlorinated solvents (e.g., dichloromethane or chloroform) and then analyzed by gas chromatography. The quantification of the methylesters by using an internal standard or a calibration curve gives the PHA content on a bacterial biomass weight basis. Despite being the method of choice in the field of PHA quantification, this procedure is far from being considered “green” (according to the modern concept of Green Analytical Chemistry, Gałuszka et al., 2013) since it is based on a lengthy transesterification reaction that needs the use of a considerable amount of harsh and harmful reagents (e.g., H_2_SO_4_ and chloroform) for forming and isolating the analytes. The need for fast routine solvent-less methods to reduce sample pretreatment, speed up the analysis and decrease the overall costs has brought toward the birth of a new generation of quantitative analysis based on the exploitation of the thermal properties of PHA (Morikawa and Marchessault, 1981; Hahn and Chang, 1995; Aoyagia et al., 2002; Li et al., 2003). PHA is thermally unstable above 180°C and this behavior can be exploited to depolymerize PHA and give specific analytes (namely 2-alkenoic acids) that can be used as markers for quantifying intracellular PHA and their monomeric composition. Thermal treatments like thermogravimetric analysis (TGA, Hahn and Chang, 1995), pyrolysis (Morikawa and Marchessault, 1981; Torri et al., 2014), or low-temperature thermolysis (Abbondanzi et al., 2017) have been exploited to this purpose, representing a faster and equally reliable alternative to conventional methanolysis-chromatography approach.

#### Molecular Weight and Polydispersity Index

Number and average molecular weight, as well as polydispersity index (a proxy of the distribution of the molecular weight and, thus, the heterogeneity of the polymer), are usually determined in chloroform solution by a comparative technique like gel permeation chromatography (GPC) using a single concentration detector (typically refractive index, RI). Care should be taken since some PHA tend to form gels in chloroform (as in some other “super-solvents” like γ-valerolactone, Samorì et al., 2016), hence their concentration should be kept below the gel point.

### General Process Steps

**FIGURE 1:**
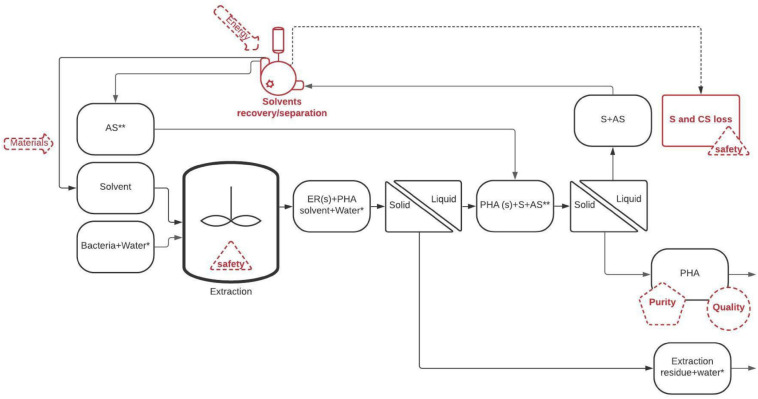
General scheme representing the solvent-based approach for recovering PHA from PHA rich bacteria. AS, anti-solvent; S, solvent; PHA, polyhdroxyalkanoate; PHA (s), polyhydroxyalkanoate in suspended solid form; ER (s), extraction residue as suspended solids. *If the extraction is performed directly on wet microbial slurry. **If an anti-solvent is used.

**FIGURE 2:**
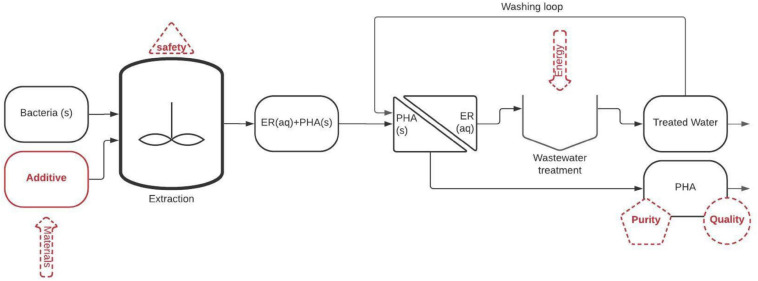
General scheme representing the cellular lysis approach for recovering PHA form PHA rich bacteria. ER (aq), extraction residue dissolved in water; PHA, polyhydroxyalkanoate; PHA (s), polyhydroxyalkanoate in suspended solid form.

The main steps involved in the two strategies (recovery with solvents and recovery by cellular lysis), as well as the most relevant technical/environmental issues that should be particularly considered (in red) are highlighted in Figures 1, 2.

Solvent extraction of PHA from dry or wet microbial biomass (e.g., microbial slurry in which the content of biomass could be around 10–20 wt%) typically includes the following steps:

(1) Putting in contact biomass and admixing it with solvent; this is a relatively low energy consumption operation.
(2) Heating up the mixture to the desired extraction temperature. Such a step involves sensible heat that can be reliably recovered (more than 80%) through counter-current heat exchangers. The order of magnitude of the energy consumption for reaching the extraction temperature could be calculated from the heat capacities of the solvent and microbial biomass. Since the extraction is typically performed at a temperature lower than the PHA decomposition temperature (<200°C), the heat capacity of solvents/biomass ranges between 1–2 kJ kg^–1^ K^–1^ and the heat capacity of water (eventually present if a microbial slurry with 10–20 wt% of biomass is subjected to the extraction) is equal to 4.2 kJ kg^–1^ K^–1^. The required energy for this operation is usually low (0.5–2 MJ kg^–1^ PHA) and it is not considered a critical issue.
(3) Separating the extraction residues (non-PHA biomass and eventually water) from the PHA-enriched phase (usually the organic solvent). This step can be accomplished through filters or other solid/liquid separations (centrifuge, settling) which typically give a PHA-enriched liquid phase (PHA dissolved in the solvent) with a low amount of residual solvent (that can be recovered up to a certain level). This step is usually favored when water-insoluble organic solvents with low viscosity are used since they can be easily separated from the aqueous slurries.
(4) Separating PHA from the solvent by evaporation of a low boiling solvent or by PHA precipitation followed by solid/liquid separation. Evaporation of the solvent can be made by distillation (providing heat) or by low-pressure evaporation (providing electrical energy). PHA precipitation can be obtained by changing the physical properties of the solvent (e.g., temperature) or by admixing it with a counter-solvent that dissolves the solvent but not PHA. Solvent evaporation is the simplest and most widely applied solution, but it represents a costly step. Thermal energy consumption is directly proportional to the Latent Heat of Evaporation (LHE, ranging from 2.3 MJ kg^–1^ for water to 0.4–1 MJ kg^–1^ for most common organic solvents and CO_2_), but “quality” of such thermal energy and therefore costs are related to the boiling temperature. This means that the organic solvents with low boiling point and low LHE are the easiest solvents to be removed.
(5) Re-obtaining of the solvent with a quality (purity) comparable with that of fresh solvent used for the extraction. This means that the solvents (or mixtures of solvent and antisolvent) should be processed to recover the largest portion in relatively clean form. For that purpose, precipitation of PHA has the unique feature to provide a direct recovery of the solvent during the separation stage. Otherwise, in most approaches both solvent and additional antisolvent can be recovered by condensation of the solvent vapors eventually assisted by a membrane separation or pervaporation (e.g., for the separation of miscible solvents). As a general rule-of-thumb, recovery by simple condensation is technically easier for compounds with high boiling points. When more complex methods, as membrane separation and pervaporation, are used to assist the solvent recovery, the efficiency is case-by-case related to the available membrane and affinity with solvents and potential interfering substances.

Due to the complexity of the process, four common/key critical issues can be identified in each solvent-based PHA extraction method:

(1) The efficiency related to solvent consumption: organic solvents cannot be 100% recovered because of leaks, losses (solvents remaining in PHA and extraction residues) and unavoidable safety needs. Even when a closed loop of solvent flow is used, a certain degree of system purging is required to avoid the formation of an explosive atmosphere.
(2) The efficiency related to the energy applied for solvent recovery: it is possible to significantly increase the solvent recovery by using higher energy and more complex equipment. The main drawback of this approach is the net energy consumption of the process as for the solvent evaporation under vacuum or the use of pervaporation through membranes.
(3) The solvent contaminations in the recovered PHA: whereas solvent extraction can be selective and could discriminate between PHA and other (polar) cellular constituents, the intrinsic affinity of the solvent for the polymer could hamper a complete solvent removal from the final product. Such aspect is quite relevant due to problems during the subsequent PHA processing (damage of injection molding equipment) and PHA utilization in the case of toxic solvents.
(4) Safety issues: all solvents but water is at least flammable and, in some cases, volatile. This means that they can accidentally form an explosive atmosphere, they can exert toxicity toward humans through respiratory systems or, being Volatile Organic Compounds (VOCs), they can cause air pollution.

The main steps involved in cellular lysis methods are presented in Figure 2.

(1) Mixing of PHA containing slurry and an additive (e.g., alkali, surfactants, or oxidants), eventually assisted by heating to improve the solubilization of non-PHA constituents. This operation produces slurry in which PHA becomes the main insoluble constituent.
(2) Separation of the slurry into a liquid solution and solid PHA usually recovered as a wet powder that must be dried and subsequently purified.
(3) Treating of the liquid solution, containing additives and solubilized bacterial biomass, as wastewater.

The main challenges of cellular lysis methods are:

(1) The soluble biomass constituents end up in the final PHA products, and, given that the dissolution process cannot be 100% efficient, the purity of the final PHA is typically the most relevant issue of such methods. This is especially important considering that impurities (e.g., endotoxins) could exert a negative effect on PHA applications (especially biomedical ones): pyrogenic endotoxin in fact causes fever if introduced into the bloodstream of humans or other animals and thus should be kept below a set limit. According to the U.S. Food and Drug Administration guideline, the upper pyrogen limit is 5.0 endotoxin units (EU)/kg (body weight) per injection.
(2) Whereas the energy input is typically lower than that necessary for solvent extractions, material consumption can be comparable or even larger than in solvent extraction methods. It follows that the recovery or saving of lytic agents through innovative approaches represents one of the challenges of the applied research concerning PHA extraction.

### Techno-Economics of the Extraction Process

The PHA extraction process is strictly ruled by operational costs (OPEX) and fixed cost (CAPEX) of the extraction apparatus like all the other industrial processes. Beyond standard solid/fluid handling systems, which give a minor contribution to the system, the extraction apparatus is constituted by functional elements with a characteristic size (e.g., volume, power, or throughput capacity) that determine the CAPEX, and consumptions (power or material consumption) that determine the OPEX.

The OPEX (€/y) of a specific extraction system with a known capacity (kgPHA/y) are the actual yearly costs due to the equipment running, like electrical energy (purchased at 0.12€/kWh) or thermal energy (purchased at 0.06 €/kWh). Such values can be calculated from basic assumptions of plant economics in chemical engineering.

The capital expenditure (CAPEX) consists of the purchasing costs of the extraction equipment (€) and increases the “financial” yearly cost depending on the maintenance costs, equipment depreciation, amortization, and interest rate. Maintenance costs are a tangible value related to the fact that any equipment requires to be maintained, and the cost of maintenance is proportional to the CAPEX. Maintenance costs are estimated at 3–7% of the CAPEX, with lower maintenance costs for mature technologies and large-scale plants, and higher maintenance costs for new technologies and small-scale equipment. Such values should be integrated with the other financial aspects (e.g., amortization over 20 years of plant operation) which add a value of 5–8% depending on the interest rate. As general rules of thumb, 1 M€ of additional CAPEX implies a yearly cost equal to 70–150 k€/y. Therefore, estimating the order of magnitude of the CAPEX of a certain extraction technology has pivotal importance for the early development stage of the technology itself. Consistent delivery of this information is not trivial and requires crossing the information from general plant economics with additional information from gray literature, namely quotations and opinion of experts working in similar fields. The scope of this review is to provide a comparison of methods from several points of view, therefore, some general rules to estimate the costs of an extraction process were drawn. This task was performed by focusing on the major (from the point of view of cost) extraction steps shown in Figures 1, 2: (i) the material input needed for the extraction (e.g., solvents or additives), (ii) the extraction reactor, (iii) the solid/liquid separator, and (iv) the solvent/water recovery/removal unit.

#### Material Input

The first relevant cost of each extraction procedure is related to the material input (additive, solvent or anti-solvent), named OPEX_*M*_ (€/kg_*PHA*_):

$$\left[O\,P\,E\,X_{M}=M_{\cos t}\cdot \frac{E\,x\,t_{r\,a\,t\,i\,o}\cdot E\,x\,t_{l\,o\,s\,s}}{X_{P\,H\,A}\cdot R_{y\,i\,e\,l\,d}}\right]$$

Where *M*_*cost*_ is the market price (€/kg) of solvents/additives used, Ext_*ratio*_ (kg_*M*_/kg_*feedstock*_) is the specific amount of material used per kg of feedstock (e.g., biomass slurry), X_*PHA*_ is the PHA content of the feedstock (kg_*PHA*_/kg_*feedstock*_), *R*_*yield*_ is the efficiency of extraction and Ext_*los*__*s*_ is the amount of material that is lost at the end of the extraction process (kg_*M*,__*lost*_/kg_*M,used*_). Ext_*los*__*s*_ ranges between 1 (e.g., a single-use surfactant) and 0 (when the solvent is completely recovered without any loss). For solvents, Ext_*loss*_ depends on leaks (usually less than 1%, Kemper, 1997) and the amount of residual solvent in the extracted product (solvent in PHA) and byproducts (solvent in the microbial residue). Overall Ext_*loss*_, which usually falls in the 0.005–0.05 range, but is deeply influenced by the chemistry of the system and process configuration.

#### Extraction Reactor

The extraction reactor consists of devices that pretreat/mix bacteria, solvents, and/or additives and manages the heating/cooling of the mixture, usually through electrical heating and a heat exchanger (since the extraction process is not significantly exo- or endothermic). The cost of the extraction reactors is mostly a function of the reactor volume (L) and relative operating pressure (bar). Being mixing almost negligible for the energy balance, the operating costs of the extraction reactor are mainly due to the heating of the mixture. Such aspects have been included in empirical relationships shown in the following equations, which can be used as a rule of thumb in the early design of new extraction processes. The CAPEX related cost (actualized considering depreciation and maintenance) of extraction vessel can be estimated as CAPEX_*extr.vessel*_ (€/kg_*PHA*_):

$$[CAPEX_{e\,x\,t\,r.v\,e\,s\,s\,e\,l}=\frac{0.1}{8760}\cdot \frac{\left(1+0.0376\,P\right)}{\sqrt{P\,H\,A_{o\,u\,t}}}\cdot 528$$

$${\left(\frac{R\,T_{e\,x\,t}\cdot \left(1+E\,x\,t_{r\,a\,t\,i\,o}\right)}{X_{P\,H\,A}\cdot R_{y\,i\,e\,l\,d}\cdot \rho_{m\,i\,x}}\right)}^{0.5}]$$

Where P is the relative pressure of extraction process (bar), PHA_*out*_ is the absolute size of the system expressed as PHA output capacity (kg_*PHA*_/h), RT_*ext*_ is the residence time in extraction vessel, Ext_*ratio*_ (kg_*M*_/kg_*feedstock*_) is the specific amount of material used per kg of feedstock (e.g., biomass slurry), X_*PHA*_ is the PHA content of the feedstock (kg_*PHA*_/kg_*feedstock*_), R_*yield*_ is the efficiency of extraction (g_*PHA, extracted*_/g_*PHA*_), and ρ_*mix*_ (kg/L)is the density of the mixture in the extraction vessel (e.g., 0.7–1.5 kg/L water and organic solvents).

The specific OPEX_*extr.vessel*_ (€/kg_*PHA*_) of the extraction reactor running is mainly related to the heating or pretreatment of the mixture as follows:

$$[OPEX_{e\,x\,t\,r.v\,e\,s\,s\,e\,l}=\frac{T\,h\,E_{p\,r\,i\,c\,e}\cdot \Delta \,T\cdot C_{p}\,\left(1+E\,x\,t_{r\,a\,t\,i\,o}\right)}{3.6\cdot 10^{6}\cdot X_{P\,H\,A}\cdot R_{y\,i\,e\,l\,d}}$$

$$+\frac{E\,l\,E_{p\,r\,i\,c\,e}\cdot E\,n_{r\,e\,q}\cdot R\,T_{e\,x\,t}\,\left(1+E\,x\,t_{r\,a\,t\,i\,o}\right)}{3.6\cdot 10^{6}\cdot X_{P\,H\,A}\cdot R_{y\,i\,e\,l\,d}\cdot \rho_{m\,i\,x}}]$$

Where ThE_*price*_ and ElE_*price*_ are respectively the thermal and electrical energy costs (€/kWh), ΔT is the difference between the inlet and outlet temperatures (equal to the reaction temperature) and minus environmental temperature if heat recovery is not applied, C_*p*_ is the specific heat capacity of extraction mixture, Ext_*ratio*_ (kg_*M*_/kg_*feedstock*_) is the specific amount of material used per kg of feedstock (e.g., biomass slurry), X_*PHA*_ is the PHA content of the feedstock (kg_*PHA*_/kg_*feedstock*_), R_*yield*_ is the efficiency of extraction, En_*req*_ is the electrical energy required per unit of volume (W L^–1^, e.g., heat dissipation, stirrers, mixers or ultrasound treatment), RT_*ext*_ is the residence time in extraction vessel, ρ_*mix*_ is the density of the mixture in the extraction vessel (e.g., 0.7–1.5 kg/L water and organic solvents). OPEX_*extr.vessel*_ is not intrinsically related to the scale of the extraction, even if En_*req*_ can be affected by the scale, with a general lower volumetric energy requirement for larger reactors.

#### Solid/Liquid Separator

A solid/liquid separation can be performed using tangential filtration, dead-end filtration (e.g., industrial filter press), or centrifugation. Each separation technique is characterized by different applicability and economic features. Tangential filtration is usually characterized by high OPEX, mainly due to recirculation pumping required, and low fixed cost, consisting of filtration elements. As a comparison, filter presses and centrifuges are characterized by high CAPEX and lower OPEX, and for this reason are the best options at a larger scale, when scale factor decreases selectively the CAPEX related cost.

The tangential filtration unit is formed by filters and recirculation pump needed for tangential low through the filters and can be used as a general model for a preliminary cost evaluation. The cost of such systems is mainly related to the CAPEX and can be easily modeled as an example technology for S/L separation:

$$[CAPEX_{S/L}=\frac{0.1}{8760}\cdot \frac{3000}{P\,H\,A_{o\,u\,t}^{0.23}}\cdot$$

$${\left(\frac{F\,i\,l\,t\,r\,a\,t\,e_{r\,a\,t\,i\,o}\cdot \left(1+F\,i\,l\,t\,r\,a\,t\,e_{w\,a\,s\,h\,l\,o\,o\,p}\right)}{F\,i\,l\,t\,e\,r_{r\,a\,t\,e}\cdot X_{P\,H\,A}\cdot R_{y\,i\,e\,l\,d}\cdot \rho_{f\,i\,l\,t\,r\,a\,t\,e}}\right)}^{0.77}]$$

Where PHA_*out*_ is the absolute size of the system expressed as PHA output capacity (kg_*PHA*_/h), Filtrate_*ratio*_ is the specific amount of external input (e.g., solvent or aqueous additive) that has to be obtained as filtrate through S/L module, Filtrate_*wash loop*_ is the times that the solid has to be washed to get an acceptable purity, Filter_*rate*_ (L/m^2^/h) is the specific filtration capacity of the filtration equipment, which depends on the filter (mainly the size of pores) and liquid viscosity, X_*PHA*_ is the PHA content of the feedstock (kg_*PHA*_/kg_*feedstock*_), R_*yield*_ is the efficiency of extraction, ρ_*filtrate*_ (kg L^–1^) is the density of filtrate. Typical values of Filter_*rate*_ in microfiltration (the most used system for separation of cells or cellular debris in aqueous slurries) is around 35 L/m^2^/h and the updated 2020 cost for microfiltration elements is around 3000 €/m^2^_*filtration area*_.

OPEX_*S/L*_ (€/kg_*PHA*_) of tangential filtration are mainly related to the recirculation pump which is about 200-folds larger than feed pumps and determines most of the energy consumption of the system:

$$\left[O\,P\,E\,X_{\frac{S}{L}}=\frac{E\,l\,E_{p\,r\,i\,c\,e}\cdot P\,W_{E,r\,e\,g}\cdot T\,F\,l\,o\,w_{r\,a\,t\,i\,o}\cdot \left(1+F\,i\,l\,t\,r\,a\,t\,e_{w\,a\,s\,h\,l\,o\,o\,p}\right)}{3.6\cdot 10^{6}\cdot X_{P\,H\,A}\cdot R_{y\,i\,e\,l\,d}\cdot \rho_{f\,i\,l\,t\,r\,a\,t\,e}}\right]$$

Where ElE_*price*_ is respectively the electrical energy price (€/kWh), PW_*E*_,_*req*_ is the work required for pumping 1 L of slurry (e.g., 360–600 J/L for large volumetric pumps working at differential pressure of 2–4 bar), TFlow_*ratio*_ is the ratio between tangential flow and filtrate flow required to avoid plugging of filters (-e.g., typically in the 100–300 range), Ext_*ratio*_ (kg_*M*_/kg_*feedstock*_) is the specific amount of material used per kg of feedstock (e.g., biomass slurry), *X*_*PHA*_ is the PHA content of the feedstock (kg_*PHA*_/kg_*feedstock*_), *R*_*yield*_ is the efficiency of extraction, ρ_*filtrate*_ (kg L^–1^) is the density of filtrate.

#### Solvent Recovery or Drying Unit (SRU)

Solvent recovery (or water removal) can be accomplished through atmospheric distillation, vacuum distillation or pervaporation through a membrane. Atmospheric distillation is characterized by high OPEX and low CAPEX, whereas vacuum distillation or pervaporation save OPEX at the expense of higher CAPEX. Despite the large variability of technologies, a base-case in which the solvent is recovered by distillation at atmospheric pressure can be considered: such conventional evaporation of organic solvents can remove a solvent from a solid material (e.g., solvent + PHA) and requires an amount of energy slightly higher than the latent heat of evaporation due to solvent losses. An important aspect is that, when distillation is applied to a solid-free mixture (e.g., solvent + anti-solvent mixture) and especially with water, multiple-effect distillation can be used, decreasing the energy requirement down to 4% of the latent heat of evaporation.

For simpler evaporators that can be used for both water evaporation and solvent recovery the CAPEX_*evaporator*_ (€/kg_*PHA*_) is intrinsically related to the amount of heat exchange required, and ultimately on the surface area of heat exchangers. Such size is proportional to the amount of heat involved in the evaporation and the number of effects used in multiple effect evaporators. The 2020 cost of evaporators with a 0.5–0.6 MW latent heat capacity (0.7–0.8 ton_*H*__2__*O*_/h) is in the 1.2–1.8 M€ range. Taking average values from quotation and, including some empirical assumptions, the CAPEX related costs associated with that step can be estimated as follows:

$$\left[C\,A\,P\,E\,X_{e\,v\,a\,p\,o\,r\,a\,t\,o\,r}=\frac{0.1}{8760}\cdot \frac{72}{P\,H\,A_{o\,u\,t}^{0.23}}\cdot \left(\frac{n_{e\,f\,f}\cdot L_{v}\cdot E\,x\,t_{r\,a\,t\,i\,o}}{3600\cdot X_{P\,H\,A}\cdot R_{y\,i\,e\,l\,d}}\right)\right]$$

Where *n*_*eff*_ is the number of effects used if multiple-effect evaporation is used (*n*_*eff*_ equal to 1 for simple drying processes), *L*_*v*_ is the latent heat of evaporation (e.g., 2.2 10^6^ J/kg for water), Ext_*ratio*_ (kg_*M*_/kg_*feedstock*_) is the specific amount of material used per kg of feedstock (e.g., biomass slurry), X_*PHA*_ is the PHA content of the feedstock (kg_*PHA*_/kg_*feedstock*_), R_*yield*_ is the efficiency of extraction. OPEX_*evaporator*_ (€/kg_*PHA*_) is described by the following equation:

$$\left[O\,P\,E\,X_{e\,v\,a\,p\,o\,r\,a\,t\,o\,r}=\frac{T\,h\,E_{p\,r\,i\,c\,e}\cdot L_{v}\cdot E\,x\,t_{r\,a\,t\,i\,o}}{3.6\cdot 10^{6}\cdot X_{P\,H\,A}\cdot R_{y\,i\,e\,l\,d}\cdot n_{e\,f\,f}}\right]$$

Where ThE_*price*_ is the thermal energy cost (€/kWh), L_*v*_ is the latent heat of evaporation (e.g., 2.2 10^6^ J/kg for water), Ext_*ratio*_ (kg_*M*_/kg_*feedstock*_) is the specific amount of material used per kg of feedstock (e.g., biomass slurry), X_*PHA*_ is the PHA content of the feedstock (kg_*PHA*_/kg_*feedstock*_), R_*yield*_ is the efficiency of extraction, n_*eff*_ is the number of effects used if multiple-effect evaporation is used (n_*eff*_ equal to 1 for simple drying processes).

Both CAPEX_*evaporator*_ and OPEX_*evaporator*_ are proportional to L_*v*_ and Ext_*ratio*_. For solvent extraction, this means that such type of costs is minimized with the use of a solvent with low L_*v*_ (e.g., weak intermolecular bonds) and high efficiency (which means lower Ext_*ratio*_). Increasing n_*eff*_ decreases the OPEX_*evaporator*_ but increases CAPEX_*evaporator*_ and this means that this parameter should be optimized according to the size of the equipment.

Applying the abovementioned formulas to some PHA extraction examples gives a general idea of the main cost issue of different extraction procedures. Nonetheless, it should be pointed out that this is just an example of a possible application of an economic analysis that can be used to evaluate the order of magnitude of costs in the infancy of process development at low Technology Readiness Level (TRL), to drive the design toward more economically sustainable processes. At high TRLs, a more detailed analysis is mandatory to provide a go-not-go decision.

**FIGURE 3:**
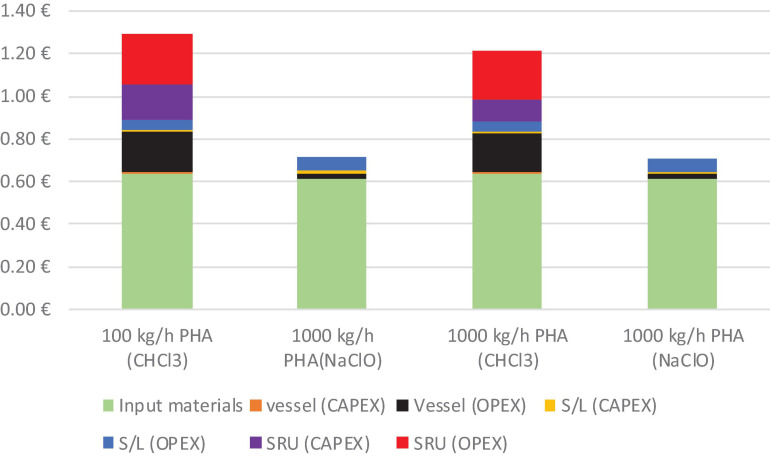
Analysis of major sources of costs of two PHA extraction processes (extraction with CHCl_3_ and cellular lysis with NaClO).

A comparison of the estimated costs for two hypothetical PHA extraction processes (extraction with chloroform at 60°C, with CHCl_3_: biomass ratio of 20:1; cellular lysis with NaClO 1.9 M at 37°C for 1 h) is reported in Figure 3. *Ex ante* evaluation of the cost of the two extraction processes gives values of 0.7–1.3 €/kg, close to the values obtained through a more detailed economic analysis performed by Fernández-Dacosta et al., 2015. Summing up this value with 0.8–1 €/kg_*PHA*_ feedstock cost (e.g., PHA from 0.4 €/kg glucose with 30–40% yield) and cost for single strain cultivation (0.8–1.8 €/kg_*PHA*_), we could calculate the PHA production cost within 2.3–4.3 €/kg_*PHA*_ range. Such data fit with a 1.4–4 €/kg_*PHA*_ realistic market price using current technologies.

This calculation highlights that cellular lysis can be considered as a low-cost extraction process that can be applied to produce low-cost and low-quality PHA even if the quite high cost of the material input. On the other hand, chlorinated solvents (characterized by the high cost of SRU) become competitive at a large scale and when PHA quality requirement prevents the use of harsh cellular lysis methods. This is in line with life cycle costing (LCC) and LCA studies focused on PHA recovery from single microbial strains reported in the literature (Saavedra del Oso et al., 2020). They reveal that:

• methods based on cellular lysis with alkaline compounds or surfactants have lower costs (1.02–5.23 €/kg_*PHA*_) than those based on solvent extraction (1.95–6.61 €/kg_*PHA*_),
• methods based on cellular lysis have better environmental performance (0.81–4.16 kg CO_2__*eq*_) than those based on solvent extraction (3.93–12.96 kg CO_2__*eq*_),
• chemicals and heat production cover more than 50% and 20–30%, respectively, of the total operating costs and are the main contributors to environmental impacts of both processes, underlying the need of reducing energy use, employing greener energy sources, and introducing chemicals recovery units where it is possible.
• PHA extraction through solvents needs large amounts of energy, especially for solvent-recovery; thus, it seems a suitable approach just for these applications in which high-quality PHA is required. However, economic and environmental performance can be optimized by employing more easily recoverable solvents, utilizing the residual heat of the PHA production plant, or process byproducts exploitable as solvents.

## Extraction of PHA From Single Microbial Strains

The research on PHA production has been focused from the beginning on the utilization of single strain cultures, and the few industrial realities that are currently producing PHA for the market are based on this approach (e.g., Biomer, Kaneka, Danimer Scientific). Different bacterial strains have been successfully used to synthesize biopolymers such as *Alcaligenes eutrophus*, *Alcaligenes latus*, *Azotobacter vinelandii*, *Azotobacter chroococcum*, *Azotobacter beijerincki*, methylotrophs, *Pseudomonas* spp., *Bacillus* spp., *Rhizobium* spp., *Nocardia* spp., and recombinant *Escherichia coli* (Pagliano et al., 2017). These bacteria can accumulate PHAs under extreme conditions such as excess carbon source and/or nutrient (nitrogen, phosphorus, sulfur, or oxygen) starvation (Reddy et al., 2003). Single strains have the double advantage to be able to accumulate a high PHA amount (above 80% of the cell dry weight) (Reis et al., 2003) and to reach high cell densities. On the other hand, such fermentations have high operating costs mainly associated with raw material and sterilization phase (Morgan-Sagastume et al., 2010).

### Solvent Extraction Methods Applied to Single Microbial Strains

#### Halogenated Solvents

Chloroform, dichloromethane, 1,2-dichloroethane, 1,1,2-trichloroethane, and 1,1,2,2-tetrachloroethane are the solvents of choice for PHA extraction since very pure polymers (more than 90%) with high molecular weight (up to 1.2 MDa) can be easily obtained (Table 1); therefore halogenated compounds are often considered as the benchmark solvents for PHA recovery (Koller et al., 2013). On the other hand, a quite wide polydispersity index range is often observed (1.75–4.50). The extraction yields seem influenced by two factors, even if a clear independent effect of all of them is difficult to be highlighted:

**TABLE 1:** PHAs extraction from single strains with solvents.

Solvent class	Solvent type and extraction conditions	PHA type and content (wt%)	Recovery (%)	Purity (%)	Molecular weight (MDa)	Polydispersity index (PDI)	References
Halogenated solvents	CHCl_3_ (25°C)	PHB (50)	27	95	1.2	–	Ramsay et al., 1994
	CHCl_3_ (61°C)	PHB (50)	55	92	0.9	–	Ramsay et al., 1994
	CHCl_3_ (61°C, acetone pretreatment)	PHB (50)	68	96	0.9	–	Ramsay et al., 1994
	CHCl_3_ (60°C)	PHB (50)	–	–	0.6–1.2	2.6–4.6	Mothes et al., 2007
	CHCl_3_ (4°C)	PHB (62)	–	–	0.8–1.0	3.1–3.7	Cavalheiro et al., 2009
	CHCl_3_ (37°C)	PHB (38)	31	92	0.8	2.6	Valappil et al., 2007
	CHCl_3_ (37°C, NaClO pretreatment)	PHB (38)	27	99	1.1	1.7	Valappil et al., 2007
	CHCl_3_ (38°C, NaClO pretreatment)	PHB (38)	30	95	0.9	3.1	Valappil et al., 2007
	CHCl_3_ (25°C, NaClO pretreatment)	PHB (65)	90–95	95	0.6	4.5	Berger et al., 1989
	CHCl_3_ and NaClO (30°C)	PHB (67)	–	97	1.0	2.0	Hahn et al., 1993
	CHCl_3_ (NaClO and NaHSO_3_ pretreatment)	PHB (55)	–	97	0.6	–	Suk Roh et al., 1995
	CHCl_3_ and NaClO (Al–, Fe-coagulants pretreatment)	PHB	90–94	98–99	–	–	Ryu et al., 2000
	C_2_H_4_Cl_2_ (83°C)	PHB (50)	54	92	0.8	–	Ramsay et al., 1994
	C_2_H_4_Cl_2_ (83°C, acetone pretreatment)	PHB (50)	69	97	0.8	–	Ramsay et al., 1994
	CH_2_Cl_2_ (rt)	P(HH-HO) (30)	86	78	–	–	Furrer et al., 2007
	CH_2_Cl_2_ and NaClO (37°C)	PHB (65)	<90	99	0.4	2.6	López-Abelairas et al., 2015
	CH_2_Cl_2_ (25°C)	PHB (50)	26	96	1.2	–	Ramsay et al., 1994
	CH_2_Cl_2_ (40°C)	PHB (50)	15	89	1.05	–	Ramsay et al., 1994
	CH_2_Cl_2_ (40°C, acetone pretreatment)	PHB (50)	24	95	1.05	–	Ramsay et al., 1994
	CH_2_Cl_2_ (60°C)	P(HB-HV) (40)	–	98	0.9–1.2	2.7–3.3	Zinn et al., 2003
	CH_2_Cl_2_	PHB (80)	–	99	–	–	Hrabak, 1992
	CH_2_Cl_2_ (35°C)	PHO (15–17)	15–20	–	0.1	3.8	Wampfler et al., 2010b
	CH_2_Cl_2_ (37°C, HCl pretreatment)	PHA	–	97	0.2–0.5	–	Martínez et al., 2011
Alkanes	Hexane (rt)	P(HH-HO) (30)	53	93	0.2	1.3	Furrer et al., 2007
	Hexane (30°C)	PHB (60)	49	>99	0.1	–	Manangan and Shawaphun, 2010
	Hexane (50°C)	PHB (98)	3	89	–	–	Aramvash et al., 2018
Alcohols	Medium chain-length alcohols	PHB (65–70)	95	>98	0.2–0.4	–	Nonato et al., 2001
	EtOH/H_2_O (30°C)	PHA (30)	96	81	0.3	2.6	Mohammadi et al., 2012a
	MeOH (50°C, NaClO pretreatment)	PHB (98)	81	99	–	–	Aramvash et al., 2018
	PrOH (100°C, NaClO pretreatment)	PHB (98)	28	97	–	–	Aramvash et al., 2018
	PrOH (rt)	P(HH-HO) (30)	23	66	–	–	Furrer et al., 2007
Esters	Butyl acetate (100°C)	P(HB-HH) (76)	42	>99	–	–	Riedel et al., 2013
	Ethyl acetate (100°C)	P(HB-HH) (76)	99	>99	–	–	Riedel et al., 2013
	Ethyl acetate (60°C)	P(HB-HH) (50)	–	–	1.0	–	Chen et al., 2001
	Butyl acetate (103°C)	PHB (7)	96	98	1.4	–	Aramvash et al., 2015
	Ethyl acetate (25°C)	PHB (7)	87	86	–	–	Aramvash et al., 2015
	Ethyl acetate (49°C)	PHB (7)	33	99	–	–	Aramvash et al., 2015
	Ethyl acetate (35°C)	PHO (15–17)	10–15	–	0.1	3.7	Wampfler et al., 2010b
	Ethyl acetate (rt)	P(HH-HO) (30)	80	92	-	-	Furrer et al., 2007
	γ- butyrolactone (120°C)	PHB (82)	45	97	0.2	2.2	Jiang et al., 2018
Carbonates	Ethylene carbonate (100°C, NaClO pretreatment)	PHB (98)	90	99	1.3	–	Aramvash et al., 2018
	1,2-propylene carbonate (130°C)	PHB (71)	95	84	0.7	3.1	Fiorese et al., 2009
	1,2-propylene carbonate (130°C, heat and pH pretreatment)	PHB (71)	95	88	1.3	3.5	Fiorese et al., 2009
	1,2-propylene carbonate (130°C)	PHA (72)	75	–	0.2	2.9	McChalicher et al., 2010
	Dimethyl carbonate (90°C)	PHB (74)	94	93	1.1	2.7	Samorì et al., 2015b
Ethers	Anisole (125°C)	PHB (58)	97	98	0.7	2.3	Rosengart et al., 2015
	Phenetole (125°C)	PHB (58)	39	–	0.5	2.2	Rosengart et al., 2015
	MTBE (35°C)	PHO (15–17)	15–20	47	0.1	3.9	Wampfler et al., 2010b
	Tetrahydrofuran (rt)	P(HH-HO) (30)	80	84	–	–	Furrer et al., 2007
Ketones	Acetone (120°C)	P(4HB-HV) (88)	92	98	1.0	1.5	Koller et al., 2013
	Acetone (22°C, NaOH pretreatment)	PHA (66)	85	78	0.08	1.8	Jiang et al., 2006
	Acetone (25°C)	PHO (60)	94	98	–	–	Elbahloul and Steinbüchel, 2009
	Acetone (30°C, NaOH pretreatment)	P(4HB) (52)	95	–	0.5	3.1	Gorenflo et al., 2001
	Acetone (rt)	P(HH-HO) (30)	83	92	–	–	Furrer et al., 2007
	Acetone (35°C)	PHO (15–17)	10–15	–	0.1	3.9	Wampfler et al., 2010b
	MIBK (100°C)	P(HB-HH) (76)	55	99	–	–	Riedel et al., 2013
	MEK (100°C)	P(HB-HH) (76)	95	>99	–	–	Riedel et al., 2013
	MEK (60°C)	P(HB-HV) (65)	94	91	0.4	2.8	Yang et al., 2015
	Cyclohexanone (125°C)	PHB (58)	93	98	0.8	1.5	Rosengart et al., 2015
	Cyclohexanone (120°C)	PHB (82)	99	99	0.2	2.1	Jiang et al., 2018
Others	DMSO (150°C)	PHB (98)	61	95	–	–	Aramvash et al., 2018
	DMF (150°C)	PHB (98)	30	97	–	–	Aramvash et al., 2018
	Acetone, EtOH and propylene carbonate	PHB (62)	85	92	1.4	–	Fei et al., 2016

*The investigated parameters are the solvents used for the extraction, the initial content of PHA in bacteria (wt% by dry weight) and the recovery (%), and the properties of the extracted PHA (purity%, molecular weight in MDa and polydispersity index).*

1. the solvent type: the recovery of PHA from single strains with a polymer amount of 50% is similar if 1,2-dichloroethane or chloroform are used under their reflux temperature, whereas the recovery with dichloromethane under the same conditions is almost four-times lower (Ramsay et al., 1990).
2. the temperature of the extraction process: Ramsay et al. (1990) demonstrated that the extraction of single strains containing 50 wt% of PHA with chloroform doubles by increasing the temperature from rt to the reflux one, but the same is not true when dichloromethane is used (results achieved at rt or under reflux are around 20%).

Many attempts have been done to improve the extraction process mediated by halogenated solvents; the application of a pre-treatment by employing chemical (e.g., treatments with oxidants, salts, or alkaline compounds) and/or physical methods (e.g., heat treatment) seems to be the best choice to improve cell disruption, promote solvent permeation and enhance the accessibility to PHAs granules. Moreover, since the high solvent to biomass ratio necessary for getting a satisfying recovery, a pre-treatment step could be necessary to reduce the amount of solvent itself, and, consequently, the costs.

If a pre-treatment of the microbial biomass with NaClO is performed before the extraction step, the PHA recovery drastically improves, especially in the case of dichloromethane (the polymer recovery is reported to increase up to 90%, López-Abelairas et al., 2015), whereas a pre-treatment with acetone does not substantially improve the results achieved without any pre-treatment (Ramsay et al., 1990). NaClO is claimed to weaken the cell membranes, thus facilitating the subsequent extraction of PHA by solvents (Koller et al., 2013). The combination of a NaClO pre-treatment and the precipitation of PHA using alcohols gives the best extraction yields and purity value (recovery up to 94% and purity up to 99%) (Berger et al., 1989; Ryu et al., 2000; López-Abelairas et al., 2015); this approach exploits the fact that PHA solubility is drastically minimized by adding a "PHA anti-solvent," typically low-molecular alcohols (ethanol or methanol), hexane, ether, acetone, or water (Koller et al., 2013), whereas impurities solubility should not be affected.

Despite being highly performing in terms of recovery yields and polymer properties, halogenated solvents are not eco-friendly, being hazardous for the environment and the operators; chloroform, for example, can affect the central nervous system and the liver, it is highly irritating for mucous membranes, the respiratory tract, and eyes, it is classified as possibly carcinogenic for humans (Group 2B, International Agency for Research on Cancer (IARC), 1999) and as Hazardous Air Pollutant, HAP (EPA Environmental Protection Agency (EPA), 1990). Additionally, halogenated solvents commonly used for PHA extraction are all fossil-based solvents. All these impactful aspects are worsened by the fact that high amounts of solvents are required for the extraction process and the large quantity of energy must be employed for the purification of the solvent and its reuse, making this approach not economically advantageous. Finally, it has been claimed that the natural morphology of the PHA granules can be affected by the use of halogenated solvents, which prevents the use of PHA for certain applications, for example in the production of strong fibers by reducing molecular mass via random and chain-end scission, particularly at a higher temperature and prolonged extraction time (Pérez-Rivero et al., 2019).

A solution to reduce the impact of these solvents on the environment and human health could be the utilization of halogen-free solvents like alkanes, alcohols, linear and cyclic esters and carbonates, ethers, ketones, organosulfur compounds, and amides (Table 1). Some of these solvents are not toxic for humans and granted GRAS (Generally Recognized As Safe) by Flavor and Extract Manufacturers Association (FEMA, Flavor and Extract Manufacturers Association), whereas others are bio-based: alcohols (ethanol and butanol) and acetone for example can be produced through biochemical approaches starting from renewable resources (e.g., alcoholic fermentations mediated by *Saccharomyces cerevisiae* or acetone–butanol–ethanol fermentation mediated by Chlostridia), whereas linear and cyclic carbonates (dimethyl carbonate or propylene carbonate) are CO_2_-derived solvents with low (eco)toxicity, complete biodegradability, and very low vapor pressure.

#### Alkanes

Alkanes used in the extraction of PHA from single strains (e.g., hexane) provide polymers with high purity (89 – ≈100%) but in low yields and with low molecular weight (about 50% and 0.2 MDa, respectively). This trend is surely due to the low solubility of PHA into such apolar solvents; on the other hand, this high lipophilicity is beneficial in terms of Gram-negative bacteria’s endotoxins abatement, providing polymers useful for medical applications, especially if an anti-solvent is used for recovering PHA (Pérez-Rivero et al., 2019): a decrease in the "endotoxicity" of PHA from 100 endotoxic units (EU g_*polymer*_-^1^) (Williams et al., 1994) to 10 EU g_*polymer*_-^1^ was achieved by using 2-propanol for precipitating PHA from *n*-hexane (Furrer et al., 2007). Despite the positive effect given by alkanes in terms of endotoxins abatement and thus health issue, alkanes cannot be considered as “green” solvents: hexane and pentane are “hazardous” compounds according to all the solvent selection guidelines published so far (Henderson et al., 2011; Prat et al., 2014, 2016), because of safety (high flammability) and environmental issues (ability to generate harmful Volatile Organic Compounds, VOC, hazardous to the atmospheric ozone layer), the last ones a common feature of longer alkanes like heptane. Therefore, the constraints on scaled-up processes based on these compounds are very strong, and their substitution during process development is a priority (Prat et al., 2016).

#### Alcohols

Contrary to alkanes, low-medium chain length alcohols are too polar solvents for being reliable candidates for PHA extraction. The fact that the extracted PHA have a low molecular weight (0.2–0.4 MDa) (Nonato et al., 2001; Mohammadi et al., 2012a) can be an indication that these polar solvents are suitable for "peculiar" polymers with short chains, presumably less difficult to be solubilized. A NaClO pre-treatment is often associated with alcohol extraction to achieve polymer purity up to 99% (Aramvash et al., 2018). As in the case of halogenated solvents, also for alcohols, the temperature affects the PHA recovery yield and purity (Mohammadi et al., 2012a): a combined synergic effect of ethanol and water has been reported by increasing the temperature from 4 to 30°C, since an increasing interaction between water and ethanol improves the cell wall breakage. Water seems to cause the release of cellular proteins and exert an osmotic pressure on the cells that result easily breakable (Mohammadi et al., 2012b). Despite alcohols seem to have a narrow range of applicability in the PHA extraction scenario, their use in scaled-up processes would be strongly encouraged: alcohols, in fact, are generally recognized as “recommended” solvents, benign in terms of acute environmental toxicity and the bio-accumulation (Henderson et al., 2011; Prat et al., 2014; Prat et al., 2016), as well in terms of health issue (butanol, in particular, is granted GRAS by FEMA and synthesizable from renewable resources thus potentially bio-based).

#### Esters

The extraction protocols based on linear and cyclic esters reported in the literature are generally performed at a quite high temperature (around 100°C); a direct comparison between ethyl and butyl acetate in the extraction of single strain bacteria containing 76 wt% of the copolymer P(HB-HH), indicates the first as the best performing among the two in terms of recovery yields, whereas the purity is excellent with both (Riedel et al., 2013). If wet biomass is extracted, a double effect of water can be observed: the hydrolysis of the esters and the reduction of the solvating power of the esters themselves (Riedel et al., 2013). Both ethyl and butyl acetate are “recommended” solvents, and all the solvent selection guides published so far agree with attributing low health and environmental scores (not problematic compounds) to the entire ester class (Henderson et al., 2011; Prat et al., 2014, 2016).

#### Carbonates

Linear and cyclic carbonates are probably among the most eco-friendly alternatives to halogenated solvents that could be applied for the extraction of PHA and PHB (the latter usually more difficult to be extracted due to its higher crystallinity and lower solubility in organic solvents), allowing the recovery of biopolymers with good properties. Dimethyl carbonate (DMC) is an example of green (it is fully biodegradable, minimally toxic for the operators and the environment) and well-performing solvent (Samorì et al., 2015a, b): DMC resulted highly efficient in terms of extraction yields, polymer purity, and polymer molecular weight, with the additional benefit of being applied directly on wet biomass. Also, 1,2-propylene carbonate (PC) and ethylene carbonate (EC), two solvents with low toxicity and therefore potentially usable in many human-related applications including cosmetics, give good results (Fiorese et al., 2009; Aramvash et al., 2018); it has been observed a decrease in the molecular weight by increasing the temperature for both solvents, probably due to a collateral reaction between the carbonate and the esters in the polymer. This suggests setting the boiling point as a limit temperature for the extraction. Despite being fewer common solvents used in industrial processes than esters and alcohols, DMC, PC, and EC are also included in the most recent solvent selection guides (Prat et al., 2016): among these polar aprotic solvents, DMC seems the greenest carbonates (ranking “recommended” because it does not have any H3xx statements after full REACh registration) and thus indicated as a potential replacement for various ketones and esters.

#### Ketones

Among ketones, only the extraction method performed by Koller et al. (2013) with acetone at high temperature and pressure (120*°*C, 7 bar) gave a polymer with high molecular weight (1.0 MDa) and PDI of 1.5, easily recoverable by cooling the solution at 4*°*C without the addition of an anti-solvent. All the other conditions/ketones work generally well in terms of recovery and purity but providing short chain length PHA. The temperature at which the extraction is performed results detrimental for recovering PHA from inside the cellular matrix: for example, cyclohexanone recovers only 16% of the polymer at 80*°*C (even after 20 h) but more than 99% within 3 min if the temperature is increased at 120*°*C (Jiang et al., 2018). In general, the ketone family has a worse profile than alcohols or esters: despite ranked as “recommended,” acetone generates VOCs, but is not toxic and readily biodegradable, whereas cyclohexanone was ranked as “problematic,” given that its synthesis via benzene and cyclohexane is not considered as sustainable (Henderson et al., 2011; Prat et al., 2014, 2016).

#### Other Solvents

Various aprotic solvents like anisole, DMSO, and DMF have been used to extract PHA from single strains. Anisole is probably the most interesting for application in a large-scale plant: PHA extraction and purity yields are over 96% (Rosengart et al., 2015), and it is ranked “recommended” since it does not have any H3xx (health hazard) or H4xx (acute environmental toxicity or bio-accumulation potential) statements (Prat et al., 2016); however, analogously to DMSO and DMF, polymer recovery has to be accomplished using an anti-solvent like ethanol, due to the boiling point above 150°C of all of them. A similar concept must be applied if non-volatile solvents like ionic liquids (IL) are used: these solvents (e.g., 1-ethyl-3-methylimidazolium diethyl- or dimethylphosphate) are capable of interacting with bacterial cell wall components like peptidoglycan and phospholipids via intermolecular H-bonds or electrostatic interactions, dissolving the NPCM and suspending PHA stored inside the cells. But since they are not removable under vacuum due to their negligible vapor pressure, alcohols are required as anti-solvents to precipitate PHA or purifying PHA after filtration (even if filtrating IL’s solution needs large porosity filters because of the intrinsic high viscosity of the solution); contrarily to what happens with high-boiling point solvents like anisole, the purity of the extracted PHA is low (from 30 to 86% if filtration or precipitation are applied, respectively) (Kobayashi et al., 2015; Dubey et al., 2018; Koller, 2020). For these reasons (low purity and “PHA-suspension” effect) the use of ILs seems more a pre-treatment than a real extraction, oriented to disaggregate the cellular components and then facilitate the action of an organic solvent capable of solubilizing PHA.

In summary, the use of solvents for the extraction of PHA from pure cultures is effective as it generally allows the extraction of PHAs with recovery yield up to 90–99% and purity yield up to 89–100%. The factors that influence mostly the extraction yields are:

• the temperature of the process, generally pivotal for increasing the solubility of PHA in the organic solvent and thus increasing the recovery efficiency;
• the nature of the polyester included inside the bacteria, being co-polymers easier to be extracted than homopolymers, especially in the case of a high percentage of monomers longer than hydroxybutyrate due to better solubility in organic solvents and a lower crystallinity,
• the amount of the polyester included inside the bacteria, since a higher number of PHA granules can exert a tensile strength on bacterial cell wall and membranes,
• the presence of water around bacterial cells (extraction of wet biomass), that can both create a barrier around the cells preventing the access of the solvent to the polymer and hydrolyze solvents (e.g., esters).

One of the main limitations in the use of solvents to extract PHA from bacterial cells relies on the need of separating the polymer from the solvent once it has been extracted; this can be done by solvent evaporation or by using an anti-solvent in which PHA is not soluble, but in both cases, a quite relevant amount of energy has to be applied. Another limitation common to both halogenated and non-halogenated solvents is the fact that a high solvent-to-biomass ratio must be used in the extraction process (up to a 20-fold mass of the microbial biomass) (Koller et al., 2013), and this is especially true for some “super-solvents.” These solvents (e.g., chloroform, γ-valerolactone, ethyl lactate, DMC) show an excellent affinity for PHA, are capable to give a concentrated liquid solution of PHA at high temperature but they become a jelly-phase (solvent entrapped in the extracted PHA) once they are at rt (Samorì et al., 2015b, 2016; Prati et al., 2019). For keeping the solutions of “super solvents” and high amounts of extracted PHA (necessary for biomass-solvent separation) as liquid, an even higher solvent-to-biomass ratio must be used. It has been claimed that the use of Soxhlet apparatus could contribute to reduce the amount of solvent necessary to achieve a satisfactory PHA recovery on a laboratory scale (Koller et al., 2013), since Soxhlet extraction is operationally a continuous-discrete technique (the solvent acts stepwise but it is also recirculated through the sample, so the system operates both in a continuous manner and as a batch system, De Castro and Priego-Capote, 2010). Some comparisons between Soxhlet extraction and direct extraction in a batch-mode underline that the PHA recovery from bacterial biomass can be higher (1.3–1.8-fold) if the first approach is applied with specific solvents like acetone, hexane, and chlorinated compounds (Jiang et al., 2006; Manangan and Shawaphun, 2010; Lupescu et al., 2016). However, a not uniform effect on the molecular weight of the recovered polymer has been observed: since bacterial samples are extracted at the solvent boiling point over long periods, thermal decomposition of thermolabile target species can occur (De Castro and Priego-Capote, 2010), especially with some solvents like chloroform (Ramsay et al., 1994), and DMC (Samorì et al., 2015a). Other solvents like acetone (Jiang et al., 2006) do not behave in this way, and no significant change in the molecular weight is observed during the Soxhlet extraction process, confirming that the temperature conditions are not in a range in which random chain-end scission can occur (Koller et al., 2013).

To the best of our knowledge, no continuous extraction protocols (e.g., countercurrent extraction or flow processes) have been applied for recovering PHA, nor at a lab-scale neither at higher TRL. However, this approach is described as the best way to obtain requirements of high safety (small volume of solvents being processed at any given time, through the elimination of large reactors), low waste generation, and energy efficiency (improvements in mixing and heating) on a large scale, pillars of the Green Chemistry and Green Engineering philosophy (Newman and Jensen, 2013). The application of continuous extraction with green solvents like carbonates, esters, and ketones capable to recover polymers with high molecular weight (1.1–1.4 MDa) and stable PDI (1.4–3.9) on a lab-scale, could be a suitable and more sustainable alternative to halogenated compounds in a scale-up perspective (Righi et al., 2017; Vogli et al., 2020).

### Cellular Lysis Methods Applied to Single Microbial Strains

The solubilization of non-PHA cellular material (NPCM) that surrounds PHA granules is an alternative approach to recover PHAs. The substances used for this purpose are oxidants like sodium hypochlorite (NaClO), alkaline or acid compounds, and surfactants (Table 2), or enzymes (Table 3).

**TABLE 2:** PHAs extraction from single strains through cellular lysis assisted by chemicals.

Chemical compound class	Chemical compound and conditions	PHA type and content (wt%)	Recovery (%)	Purity (%)	Molecular weight (MDa)	Polydispersity index (PDI)	References
Oxidants	NaClO (25°C, 1.9 M)	PHB (65)	87	93	0.5–0.8	2.4	Heinrich et al., 2012
	NaClO (37°C, 1.9 M)	PHB (65)	>80	99	0.25	6.8	López-Abelairas et al., 2015
Alkali	NaOH (30°C, 0.2 M)	PHB (77)	90	91	1.9	2.7	Choi and Lee, 1999
	KOH (30°C, 0.1 M)	PHB (77)	93	92	2.0	2.9	Choi and Lee, 1999
	NH_4_OH (30°C, 0.1 M)	PHB (77)	95	85	–	–	Choi and Lee, 1999
	NH_4_OH (90°C, 0.1 M)	P(HB-HV)	75	70	0.7	2.2	Samorì et al., 2015b
	NaOH (4°C, 0.05 M)	PHA (30)	97	97	0.15	–	Mohammadi et al., 2012b
	NaOH (30°C, 0.1 M)	P(HB-HH)	90	80	–	–	Anis et al., 2013a
	NaOH (60°C, 1 M)	PHA (70)	45	78	–	–	Yang et al., 2011
	NaOH (37°C, 0.5 M)	PHB (65)	<80	>90	0.3	2.9	López-Abelairas et al., 2015
	NaOH (30°C, NaCl pretreatment)	P(HB-HH) (68)	98	97.5	0.3	2.3–2.5	Anis et al., 2013b
Acids	HCl (30°C)	PHB (77)	95	79	–	–	Choi and Lee, 1999
	H_2_SO_4_ (30°C)	PHB (77)	96	79	–	–	Choi and Lee, 1999
	H_2_SO_4_ (80°C)	PHB (65)	80	99	0.4	3.0	López-Abelairas et al., 2015
	H_2_SO_4_ (121°C, 0.1 M)	PHB (61)	99	98	0.06	3.2	Yu and Chen, 2006
	Acetic acid (100°C, NaClO pretreatment)	PHB (98)	37	97	–	–	Aramvash et al., 2018
Surfactants	Lysol and NaOH	P(3HB-4HB)	98	97	0.4	2.9	Irdahayu et al., 2017
	CTAB (30°C)	PHB (77)	93	84	–	–	Choi and Lee, 1999
	Palmitoyl Carnitine (30°C)		Degree of lysis: 56–78%				Lee et al., 1993
	SDS	PHA (70)	81	90	–	–	Yang et al., 2011
	SDS (30°C)	PHB	90	95	0.5	–	Kim et al., 2003
	SDS (25°C)	PHB (50)	–	87	0.8	3–6	Ramsay et al., 1990)
	SDS (25°C, NaClO pretreatment)	PHB (50)	–	98	0.7	2–7	Ramsay et al., 1990
	SDS (30°C)	PHB (77)	87	98	–	–	Choi and Lee, 1999
	SDS (55°C, NaClO pretreatment)	PHA (75)	87	98	–	–	Dong and Xuenan, 2000
	SDS (50°C, autoclave pretreatment)	PHA (12)	–	95	0.2	–	Strazzullo et al., 2008
	SDS (90°C)	P(HB-HV)	100	99	1.2	2.2	Samorì et al., 2015b
	NH_4_-laurate (90°C)	P(HB-HV)	100	98	0.6	1.9	Samorì et al., 2015b
	Sodium polyoxoethylene sulfate (ES702)	PHA (70)	85	90	–	–	Yang et al., 2011
	Sodium alpha olefin sulfonate (AOS-40)	PHA (70)	87	91	–		Yang et al., 2011
	Dioctylsulfosuccinate sodium salt (30°C)	PHB (77)	93	86	–	–	Choi and Lee, 1999
	Linear alkylbenzene sulfonic acid (LAS-99)	PHA (70)	87	86	–	–	Yang et al., 2011
	Tween 20 (30°C)	PHB (77)	92	80	–	–	Choi and Lee, 1999
	Triton X-100 (30°C)	PHB (77)	93	80	–	–	Choi and Lee, 1999
	Triton X-100 (25°C)	PHB (50)	–	87	0.8	3–6	Ramsay et al., 1990
	Triton X-100 (25°C, NaClO pretreatment)	PHB (50)	–	98	0.8	2–7	Ramsay et al., 1990
	Betaine + EDTA (50°C)	PHB (70%)			0.3–0.4	–	Chen et al., 1999

*The investigated parameters are the chemicals used for the cellular lysis, the initial content of PHA in bacteria (wt% by dry weight) and the recovery (%), and the properties of the extracted PHA (purity%, molecular weight in MDa and polydispersity index).*

**TABLE 3:** PHAs extraction from single strains through cellular lysis assisted by enzymes.

Enzyme class	Extraction conditions	PHA type and content (wt%)	SNPHA (%)	Recovery (%)	Purity (%)	Molecular weight (MDa)	Polydispersity index (PDI)	References
Proteases	Trypsin (50°C, heat pretreatment at 85°C)	PHB (75)^*a*^	60	88	53	0.63	–	Kapritchkoff et al., 2006
	Bromelain (50°C, heat pretreatment at 85°C)	PHB (75)^*a*^	67	59	89	0.53	–	Kapritchkoff et al., 2006
	Bromelain and Trypsin (heat pretreatment at 85°C)	PHB (75)^*a*^	64	89	57	–	–	Kapritchkoff et al., 2006
	Trypsin and Bromelain (heat pretreatment at 85°C)	PHB (75)^*a*^	73	91	83	0.55	–	Kapritchkoff et al., 2006
	Bromelain (twice) (heat pretreatment at 85°C)	PHB (75)^*a*^	78	90	88	0.58	–	Kapritchkoff et al., 2006
	Pancreatin (50°C, heat pretreatment at 85°C)	PHB (75)^*a*^	–	90	62	–	–	Kapritchkoff et al., 2006
	Enzymes from *A. oryzae* (48°C, heat pretreatment at 85°C)	P(HB-HV) (79)^*b*^	89	–	99	0.13	2.1	Kachrimanidou et al., 2016
	Protease (37°C)	PHB (90)^*b*^	–	45	97	1.4	–	Divyashree et al., 2009
	*Microbispora* culture filtrate (50°C, heat pretreatment at 85°C)	P(HB-HV) (50)^*a*^	–	21	90	–	–	Lakshman and Shamala, 2006
	*Microbispora* culture filtrate (50°C, heat pretreatment at 85°C)	P(HB-HV) (50)^*b*^	–	49	90	–	–	Lakshman and Shamala, 2006
	Alcalase (70–50°C, heat pretreatment at 135°C)	P(HB-HV) (71)^*b*^	53	84	–	–	–	Holmes and Lim, 1990
	Alcalase (twice, 55°C, heat pretreatment at 135°C)	P(HB-HV) (71)^*b*^	79	92	–	–	–	Holmes and Lim, 1990
	Alcalase and SDS (heat pretreatment at 135°C)	P(HB-HV) (71)^*b*^	63	87	–	–	–	Holmes and Lim, 1990
	Alcalase, SDS and H_2_O_2_ (80°C, heat pretreatment at 135°C)	P(HB-HV) (71)^*b*^	90	96	–	–	–	Holmes and Lim, 1990
	Alcalase, SDS and H_2_O_2_ (80°C, heat pretreatment at 135°C)	P(HB-HV) (71)^*b*^	95	98	–	–	–	Holmes and Lim, 1990
	Alcalase and Neutrase (70°C), Protease L330 (75°C), then SDS (100°C) (heat pretreatment at 150°C)	P(HB-HV) (75)^*a*^	77	93	–	–	–	Holmes and Lim, 1990
	Esperase and SDS (heat pretreatment at 85°C)	PHB^*c*^	82	–	71	–	–	Suzuki et al., 2008
	Alcalase and SDS (55°C), then EDTA and lysozyme (30°C) (heat pretreatment at 120°C)	PHA (34)^*c*^	–	–	93	–	–	Yasotha et al., 2006
	Alcalase and SDS (55°C), then EDTA and lysozyme (30°C) (heat pretreatment at 120°C)	PHA^*a*^	–	–	97	0.06	1.87	Kathiraser et al., 2007
	Alcalase (55°C), SDS (60°C), heat pretreatment at 120°C)	PHA (30-60)^*a*^	–	98	92	–	–	De Koning and Witholt, 1997
	Alcalase (55°C) and Lecitase 100S (40°C), heat pretreatment at 100°C)	PHB (52)^*b*^	75	88	–	–	–	Holmes and Lim, 1990
Phospholipases	Lecitase 100S (40°C, heat pretreatment at 100°C)	PHB (52)^*b*^	27	65	–	–	–	Holmes and Lim, 1990
Glycosidases	Lysozyme (37°C, heat pretreatment at 85°C)	PHB (75)^*a*^	26	–	20	0.55	–	Kapritchkoff et al., 2006
	Celumax (70°C, heat pretreatment at 120°C)	P(HB-HV) (75)^*a*^	–	93	94	–	–	Neves and Müller, 2012
	Celumax (60°C, heat pretreatment at 120°C)	P(HB-HV) (75)^*a*^	–	86	–	–	–	Neves and Müller, 2012

*The investigated parameters are the enzymes used for the cellular lysis, the initial content of PHA in bacteria (wt% by dry weight), the non-PHA biomass solubilization (SNPHA) and the recovery (%), and the properties of the extracted PHA (purity%, molecular weight in MDa and polydispersity index). ^*a*^PHA recovered by solvent extraction. ^*b*^PHA recovered by centrifugation. ^*c*^PHA recovered by ultrafiltration/diafiltration.*

#### Oxidants

NaClO is a strong oxidizing chemical able to degrade proteins, lipids, carbohydrates, and nucleic acids that constitute the non-PHA matter, thus enhancing their solubilization in aqueous solutions. Although PHA with a purity up to 99% and a recovery yield over 80% can be obtained (Heinrich et al., 2012; López-Abelairas et al., 2015), the treatment with this salt drastically reduces the molecular weight of the polymer. A high amount of NaClO solution is usually required, making this method appropriate only to enhance recovery yield (Hahn et al., 1993). Besides, the application of NaClO for NCPM digestion always bears the risk of formation of toxic halogenated compounds, and it appears rather difficult to completely remove NaClO traces from the recovered PHA (Koller et al., 2013).

#### Alkaline and Acid Compounds

Sodium hydroxide (NaOH) and potassium hydroxide (KOH) are milder digestion agents that cause saponification of the lipids present in the cell wall of microorganisms, increasing membrane permeability and helping the release of proteins and non-PHA material (Kosseva and Rusbandi, 2018). This kind of treatment provides a very high polymer recovery and purity (above 90%), even under relatively mild conditions (0.1–0.2 M at 30°C); more important, the molecular weight of the extracted PHA seems to be not affected by alkali, being around 1.9–2.0 MDa with a polydispersity of 2.70–2.90 (Choi and Lee, 1999), probably due to the limited digestion time (1 h) performed at low temperature (30°C) (Mannina et al., 2020). In the literature, various acids like H_2_SO_4_ or HCl were studied for PHAs recovery, providing high recoveries (above 90%), purity (up to 99%) but low molecular weight (around 0.06 MDa) (Yu and Chen, 2006). Furthermore, the potential hazard and corrosivity of these chemicals cause risks for the operators when they are used in high concentrations, making their use inappropriate for industrial applications (Yu and Chen, 2006).

#### Surfactants

Surfactants’ mode of action is based on first incorporation into the lipidic bilayer of cell membranes, followed by an increase of the cellular volume until the membrane breaks to produce large micelles of surfactant and phospholipids. This finally causes the PHA release (Pérez-Rivero et al., 2019). Furthermore, surfactants can solubilize proteins and other non-PHA cellular material facilitating the disruption of cell membranes (Mannina et al., 2020). Anionic surfactants like sodium dodecyl sulfate (SDS) or alkylbenzene sulfonates (LAS) are the most used surfactants, tested alone or in combination with chemical or thermal pretreatments; however, SDS seems to give random results in terms of recovery, purity, molecular weight, and polydispersity index, and it is not easy to attribute its real effect on all of these parameters. Cationic surfactants like benzalkonium chlorides (BAC), palmitoyl carnitine, or hexadecyltrimethylammonium bromide (CTAB) are less used compounds than anionic ones and seem to decrease the molecular weight of the recovered polymer (despite the purity and the recovery are similar). Non-ionic surfactants like Triton X-100 or Tween-20 perform well in terms of polymer recovery, polymer purity, and molecular weight of the recovered polymer.

Surfactants are often combined with NaOH, NaClO, and chelating agents to increase the purity and recovery yield of the extracted polymer (Ramsay et al., 1990; Dong and Xuenan, 2000). Degradation of polymer granules and therefore reduction of molecular weight can generally occur only if high temperatures or pH are used (Kunasundari and Sudesh, 2011). The main limitation of this approach is the high dosage of highly water-soluble surfactants that have to be used for achieving satisfactory PHA recovery; this results in high costs of recovery and treatment of wastewater (Kapritchkoff et al., 2006). To overcome this drawback, the application of recoverable and recyclable surfactants can bring an overall improvement of the process, both in terms of economics and environmental footprint; for example, Switchable Anionic Surfactants (SAS) are compounds whose water solubility can be tuned by the addition and removal of CO_2_ (Samorì et al., 2015b): cellular lysis is accomplished by using the water-soluble (ionic) form of the surfactant, whereas the recovery (more than 90%) of the surfactant in its non-ionic form is achievable by changing the pH of the aqueous solution using CO_2_. In this way, even if high doses of surfactants are necessary to achieve an excellent polymer recovery, the surfactant can be completely recycled.

#### Enzymes

Specific enzymes like proteases and glycosidases can be applied to PHA-producing bacteria to hydrolyze peptide or glycosidic bonds of microbial proteins and carbohydrates/complex carbohydrates (e.g., glycoproteins and glycolipids), respectively. Alcalase (subtilisin A) is one of the most used proteases applied for PHA recovery, thanks to very broad substrate specificity and a good tolerance toward a quite large working temperature range (45–65°C); it usually provides recoveries above 90%. Other proteases like Trypsin and Bromelain work well in terms of polymer recovery (around 90%) and molecular weight (0.5–0.6 MDa); the non-PHA biomass solubilization (SNPHA) is generally higher with this class of enzymes than with glycosidases or phospholipases, ranging between 53–95% vs. about 26%. To increase the extraction efficiency, cocktails of proteases, nucleases, phospholipases, lysozymes, and other enzymes, in combination with surfactants and chelating agents can be applied. Centrifugation and use of solvents are the most employed extraction method but ultrafiltration and diafiltration are also applied (Kapritchkoff et al., 2006; Kathiraser et al., 2007); if the purity of the recovered polymer is similar, simple centrifugation seems to provide higher recovery than the use of organic solvents (Lakshman and Shamala, 2006).

In summary, enzymatic processes allow extraction of PHA with a wide range of purity (up to 88.8–97%) because the enzyme lysis effect is strictly related to reactions they catalyze. However, the process is expensive due to the high cost of enzymes and the complexity of the extraction process (Patel et al., 2009).

An alternative cell lysis approach recently described to recover PHA from single strains exploits whole microorganisms as cell-lytic agents (namely predatory bacteria of other Gram-negative bacteria), instead of isolated enzymes (Koller, 2020); these predatory bacteria (e.g., the species *Bdellovibrio bacteriovorus*) have a hydrolytic arsenal (e.g., extracellular-like PHA depolymerase) capable of breaking the cell walls of the prey (the culture of PHA-producing bacteria) in a relatively short time frame (24 h of “predation”), causing the release of the intracellular PHA in the culture medium (Martínez et al., 2016). To avoid the further hydrolysis of the released PHA granules by extracellular-like PHA depolymerase (that can hydrolyze up to 80% of the PHA produced by the prey), engineering of the predator seems to be the method of choice (Martínez et al., 2016). This process has been proposed as robust and generalizable since applicable to prey upon a wide range of Gram-negative bacteria, however, it is worth mentioning that high PHA purity values can be achieved just after extraction of the co-culture’s sediments with chlorinated solvents. A strictly related approach (even if on a longer time frame) is the use of some animals like the mealworm *Tenebrio molitor* (Murugan et al., 2016) or rats (Kunasundari et al., 2013; Ong et al., 2018) to digest the NPCM of PHA-producing bacteria and excrete PHA granules with a high purity level, intact molecular weight and native morphology. Even in this case, PHA must be separated from the fecal pellets with chemicals (e.g., alkaline water), thus these approaches could be considered as low-cost and sustainable pre-treatments instead of self-sustaining recovery methods.

## Extraction of PHA From Mixed Microbial cultures

Mixed microbial cultures (MMC), able to accumulate intracellular PHA similarly to what occurs with single strains and with comparable production rates, are probably the new frontier in the field of marketable PHA production, especially if this activity is coupled with wastewater treatment for reducing the cost of raw material and energy consumption aspects (Dias et al., 2006; Jiang et al., 2018). However, MMC downstream costs can be even more impacting on the overall process than single strain downstream costs especially because of a more challenging PHA extraction phase (Patel et al., 2009; Samorì et al., 2015a) and the difficulty in extracting PHA from MMC can be reflected both in terms of polymer recovery and purity (Mannina et al., 2019).

### Solvent Extraction Methods Applied to Mixed Microbial Cultures

#### Halogenated Solvents

Chloroform and dichloromethane are the most used solvents applied so far in the extraction of PHA from MMC (Table 4). In some cases, an acetone pre-treatment was used to enhance cell breakage and improve the recovery of PHA (Serafim et al., 2008b); PHA recovery is usually achieved by solvent evaporation or by polymer precipitation with water, alcohols (methanol and ethanol) or cold petroleum ether. Occasionally a subsequent purification step, which consists of a washing step with acetone and diethyl ether, has been applied.

**TABLE 4:** PHAs extraction from mixed microbial cultures with solvents.

Solvent class	Solvent type and extraction conditions	PHA type and content (wt%)	Recovery (%)	Purity (%)	Molecular weight (MDa)	Polydispersity index (PDI)	References
Halogenated solvents	CH_2_Cl_2_ (50°)	P(HB-HV) (40)	52	94	1.4	2.0	Samorì et al., 2015a
	CH_2_Cl_2_ (acetone pretreatment)	P(HB-HV) (25)	18–30	–	2-3	1.3	Patel et al., 2009
	CH_2_Cl_2_	PHB (72)	56	98	1.8	–	Jiang et al., 2015
	CHCl_3_ (37°C, HCl pretreatment)	P(HB-HV) (40–65)	–	–	0.2–0.4	1.3–1.7	Duque et al., 2014
	CHCl_3_ (100°C)	PHA	–	–	0.3–0.9	1.7–3.9	Bengtsson et al., 2010
	CHCl_3_ (100°C)	P(HB-HV) (25)	–	–	0.3–1.0	1.5–2.5	Morgan-Sagastume et al., 2010
	CHCl_3_ (37°C)	P(HB-HV) (65–72)	–	–	0.2–0.6	2.3–2.7	Albuquerque et al., 2011
	CHCl_3_	P(HB-HV) (28)	–	–	0.1–0.4	2.2–3.7	Dai et al., 2007
	CHCl_3_ (4°C)	PHA	–	–	0.4–0.6	1.4–1.7	Dai et al., 2008
	CHCl_3_ (reflux)	PHB (60)	–	–	0.5	–	Dobroth et al., 2011
	CHCl_3_ (100°C, acidic MeOH and benzoic acid pre-treatment)	P(HB-HV)	–	–	0.1–0.9	1–6	Lemos et al., 1998
	CHCl_3_	PHB (66)	–	–	2.1–3.4	1.3–2.2	Serafim et al., 2008b
	CHCl_3_ (reflux, acetone pretreatment)	PHB	–	–	0.2–0.4	1.2–2.7	Hu et al., 2013
	C_2_H_4_Cl_2_ (100°C, HCl and PrOH)	PHB (89)	–	–	–	–	Johnson et al., 2009
Alcohols	Butanol (125°C)	P(HB-HV) (15)	–	95–98	0.4–0.6	1.8–3.4	Laycock et al., 2014
	2-butanol (125°C)	PHB (44)	83	–	0.7	–	Werker et al., 2015
Carbonates	Dimethyl carbonate (90°C)	P(HB-HV) (40)	49	98	1.3	1.9	Samorì et al., 2015a
	Dimethyl carbonate (soxhlet)	P(HB-HV) (40)	41	87	0.5	2.9	Samorì et al., 2015a
	Dimethyl carbonate (90°C, NaClO pretreatment for 5 min at rt)	P(HB-HV) (40)	40	92	0.6	2.6	Samorì et al., 2015a
	Dimethyl carbonate (90°C, NaClO pretreatment for 15 min at rt)	P(HB-HV) (40)	62	98	0.8	2.4	Samorì et al., 2015a
	Dimethyl carbonate (90°C, NaClO pretreatment for 1h at rt)	P(HB-HV) (40)	76	88	0.6	2.3	Samorì et al., 2015a
	Dimethyl carbonate (90°C, NaClO pretreatment for 5 min at 100°C)	P(HB-HV) (40)	72	89	0.5	2.8	Samorì et al., 2015a
	Dimethyl carbonate (90°C, NaClO pretreatment for 15 min at 100°C)	P(HB-HV) (40)	72	92	0.5	2.4	Samorì et al., 2015b
	Dimethyl carbonate (90°C, NaClO pretreatment for 1h at 100°C)	P(HB-HV) (40)	82	93	0.2	2.5	Samorì et al., 2015a
	Dimethyl carbonate (90°C, twice)	P(HB-HV) (40)	12	95	1.2	2.7	Samorì et al., 2015a
Ketones	Acetone (125°C)	P(HB-HV) (15)	–	95–98	0.4–0.6	1.9–3.4	Arcos-Hernández et al., 2013
	Acetone (125°C)	P(HB-HV) (15)	–	95–99	0.5–0.6	1.9–3.0	Laycock et al., 2014
	Acetone (125°C)	P(HB-HV)	51	75–98	0.2–0.6	1.7 ± 0.1	Chan et al., 2017

*The investigated parameters are the solvents used for the extraction, the initial content of PHA in bacteria (wt% by dry weight) and the recovery (%), and the properties of the extracted PHA (purity%, molecular weight in MDa and polydispersity index).*

Patel et al. (2009) obtained an ‘ultra-high molecular weight PHAs’ (2–3 MDa) with a very narrow size distribution (PDI of 1.3) by applying dichloromethane; however, the recovery was very low (18–30%), suggesting that such a low result could be due to the presence of a PHA “difficult to be extracted” or to the presence of extracellular biomass matrix in which the PHA containing cells are embedded (Patel et al., 2009). This observation was confirmed by Samorì et al. (2015a), with a polymer recovery of 52%, a polymer molecular weight of 1.4 MDa, and a polydispersity of 2.0. Generally, chloroform allows the recovery of polymers with a lower molecular weight if compared to dichloromethane (Serafim et al., 2008b) (Table 4).

#### Halogen-Free Solvents

The use of non-conventional solvents for extracting PHA from MMC is not so well documented as in the case of single strains; in particular, the presence of longer co-monomers than 3HB can influence the extraction thanks to a change in the polarity and the crystallinity of the polymer (Laycock et al., 2014).

Alcohols like butanol and ketones like acetone allowed the recovery of PHAs with a purity up to 98% but the molecular weight dropped to 0.2–0.6 MDa with a polydispersity of 1.7–3.4 (Laycock et al., 2014).

Carbonates like dimethyl carbonate provide a recovery percentage of about 60% over two cycles, with a molecular weight above 1 MDa and a high purity level (98%) (Samorì et al., 2015a). If a NaClO pre-treatment is coupled to DMC extraction, the overall recovery process increases up to 82% but the molecular weight drops down by five times (0.2 MDa): reaction time and temperature of this chemical pre-treatment strongly influence the molecular weight of the recovered polymers because of the oxidizing properties and nucleophilicity of NaClO. The degrading effect on the polyesters is also emphasized by the increase in the PDI values, a sign of the polymer chains shortening due to some random chain scission.

### Cellular Lysis Methods Applied to Mixed Microbial Cultures

The chemicals mainly used for the extraction of PHA from MMC through cellular lysis are oxidants, alkali, and surfactants, similarly to what is usually applied to single strains (Table 5). On the other hand, the use of enzymes to solubilize the non-PHA cellular matrix is not reported, presumably because the enzymatic digestion is "too mild" for the high cellular resistance of MMC.

**TABLE 5:** PHAs extraction from mixed microbial cultures through cellular lysis assisted by chemicals.

Chemical compound class	Chemical compound and conditions	PHA type and content (wt%)	Recovery (%)	Purity (%)	Molecular weight (MDa)	Polydispersity index (PDI)	References
Oxidants	NaClO (85°C)	P(HB-HV) (54)	50	86	0.1	2.2–3.2	Mannina et al., 2019
	NaClO (100°C)	P(HB-HV) (40)	79	77	0.3	2.7	Samorì et al., 2015a
	NaClO (rt, 3 h)	P(HB-HV) (46)	100	90	0.3–0.5	4–10	Villano et al., 2014
	NaClO (rt, 24 h)	P(HB-HV) (46)	100	90	0.3–0.5	4–10	Villano et al., 2014
Surfactants	Ammonium Laurate (75°C, NaClO pretreatment)	P(HB-HV) (54)	74?	>99	0.1	2.2–3.2	Mannina et al., 2019
	Ammonium Laurate (75°C)	P(HB-HV) (54)	80	48	0.1	2.2–3.2	Mannina et al., 2019
	SDS (75°C, NaClO pretreatment)	P(HB-HV) (54)	59	92	0.1	2.2–3.2	Mannina et al., 2019
	SDS (75°C)	P(HB-HV) (54)	83	42	0.1	2.2–3.2	Mannina et al., 2019
	SDS (30°C)	PHB (68)	63	79	–	–	Jiang et al., 2015
	SDS (30°C, freeze-dried pretreatment)	PHB (70)	94	93	–	–	Jiang et al., 2015
	SDS (rt)	P(HB-HV) (50)	67	56	–	–	Samorì et al., 2015a
							Jiang et al., 2015
Alkali	NaOH (30°C, 1 h)	PHB (69)	97	87	0.3	–	Jiang et al., 2015
	NaOH (30°C, 0.5 h)	PHB (69)	98	89	0.3	–	Jiang et al., 2015
	NaOH (30°C, 1 h, lyophilization and freezing pretreatment)	PHB (70)	95	96	0.3	–	Jiang et al., 2015
	NaOH (rt, 3 h)	P(HB-HV) (46)	87	90	0.3–0.5	4–10	Villano et al., 2014
	NaOH (rt, 24 h)	P(HB-HV) (46)	80	91	0.3–0.5	4–10	Villano et al., 2014
	NaOH (30°C, 1 h, SDS)	PHB (66)	91	99	0.5	–	Jiang et al., 2015
	NH_4_OH (30°C, 1 h)	PHB (69)	63	63	–	–	Jiang et al., 2015
	NH_4_OH (30°C, 1 h, lyophilization and freezing pretreatment)	PHB (70)	95	87	–	–	Jiang et al., 2015
	NH_4_OH (75°C, 3 h, NaClO pretreatment)	P(HB-HV) (54)	59	97	0.1	2.2–3.2	Mannina et al., 2019
	NH_4_OH (75°C, 3 h)	P(HB-HV) (54)	73	47	0.1	2.2–3.2	Mannina et al., 2019

*The investigated parameters are the chemicals used for the cellular lysis, the initial content of PHA in bacteria (wt% by dry weight) and the recovery (%), and the properties of the extracted PHA (purity%, molecular weight in MDa and polydispersity index).*

#### Oxidants

Analogously to single strains (see section “Cellular lysis methods applied to single microbial strains”), NaClO can degrade most of the NPCM (it becomes water-soluble when oxidized) of MMC, while the PHA granules can be easily separated by precipitation (Mannina et al., 2020). However, also in this case, the treatment with NaClO can affect the quality of the obtained PHA (in terms of purity, polydispersity index, and molecular weight), compromising the final polymer applications (Kunasundari and Sudesh, 2011; Samorì et al., 2015a; Mannina et al., 2019). Villano et al. (2014) treated MMC with NaClO for 3 and 24 h at rt and noticed that the effect of NaClO on the purity and the molecular weight of the extracted polymer is similar regardless of the reaction time; on the other hand, the temperature of the NaClO treatment inversely impacts both the recovery and the polydispersity index: the PHA recovery decreases from rt (100%) to 85°C (50%), whereas the polydispersity index increases (giving more heterogeneous polymers) if the treatment is performed at high temperature (values around 3) or rt (values of 4–10) (Table 5). Recently, Lorini et al. (2020) investigated the impact of two stabilization protocols applied at the end of the accumulation phase (thermal drying at 145°C for 30 min and at 70°C overnight, and wet acidification with H_2_SO_4_) in combination with NaClO digestion (at rt overnight) for preserving the amount and the length of PHA produced by MMC. A wet-acidification pretreatment provides three-times higher molecular weights (0.4 MDa) than a thermal pre-treatment, a recovery of 96%, and a polymer purity of 90%.

#### Alkaline Compounds

According to Jiang et al. (2015), alkaline treatments cause PHA hydrolysis and the consequent decline of the molecular weight (generally between 0.3 and 0.5 MDa) and increase of the polydispersity index, but if a pretreatment like freeze-drying has applied this phenomenon is mitigated. A possible explanation relies on the fact that PHA crystallization can increase the resistance toward chemical treatments induced either by complete removal of water or by damaging the cell membrane through surfactants (Jiang et al., 2015). Among the tested alkaline compounds, NaOH gives higher PHA recovery, purity, and molecular weight than NH_4_OH, but also higher polydispersity index values (4–10 *vs.* 2–3) (Jiang et al., 2015; Mannina et al., 2019). However, NH_4_OH could be a better alternative than NaOH since it is claimed to be potentially easier to be recycled (Mannina et al., 2019); additionally, NH_4_OH performances can be improved by applying specific pretreatments (Jiang et al., 2015): if a NaClO pretreatment is applied, the polymer purity increases, whereas if a freeze-drying pretreatment is applied the polymer recovery increases (Table 5). Recently Burniol-Figols et al. (2020) investigated the effect of NH_4_OH on PHA purity, recovery, and molecular weight: long incubation times and high NH_4_OH concentrations negatively impact the polymer recovery but have no influence on its purity, whereas increasing the temperature of the digestion both purity and recovery increase. However, even if at 140°C the highest recovery and purity is achieved (90 and 83%, respectively), a drastic reduction of the molecular weight is also observed (from 0.6 MDa at 30°C to 0.08 MDa at 140°C), suggesting an optimal temperature range of 75–115°C to get high recovery of PHA (above 90%) and maintaining the molecular weight of PHA at reasonable values (0.2 MDa).

A direct comparison between NaOH and NaClO treatments underlines that NaClO works better under the same conditions (Villano et al., 2014) and the PHA recovered using NaClO is more thermally stable (up to 200°C) than that recovered by NaOH (between rt and 100°C) (Laycock et al., 2014).

#### Surfactants

Surfactants seem to be an effective way to extract PHA with good purity and in a satisfactory amount just in combination with specific pre-treatments (Table 5). For example, if SDS or ammonium laurate are applied alone, purity values of 40–60% are obtained, whereas if NaClO or freeze-drying are applied as pre-treatments before the use of the surfactant, this parameter increases up to 90%. However, surfactants have an impact on PHAs molecular weight (around 0.1 MDa), independently by the pre-treatment (Mannina et al., 2019). Furthermore, a high surfactant to biomass ratio is usually required, and consequently, the surfactant recovery generates a large quantity of wastewater that has to be treated (Jacquel et al., 2008); for overcoming this issue, as for single strains, recyclable surfactants like Switchable Anionic Surfactant (SAS) have been applied on MMC by Mannina et al. (2019): ammonium laurate gives a polymer purity of 48%, improvable by a pre-treatment with NaClO (100%). Also in this case, the molecular weight of extracted polymers is quite low (0.1 MDa) and the polydispersity is 2.2–3.2 (Mannina et al., 2019) (Table 5), but the surfactant proved to be completely recyclable.

## Overall Comparison Between the Two Extraction Approaches

The overview of the available extraction methods reported in Tables 1–5 allows a comparison of the performance on single strains and mixed microbial cultures in terms of the overall efficiency of the process of PHA recovery.

### Recovery

**FIGURE 4:**
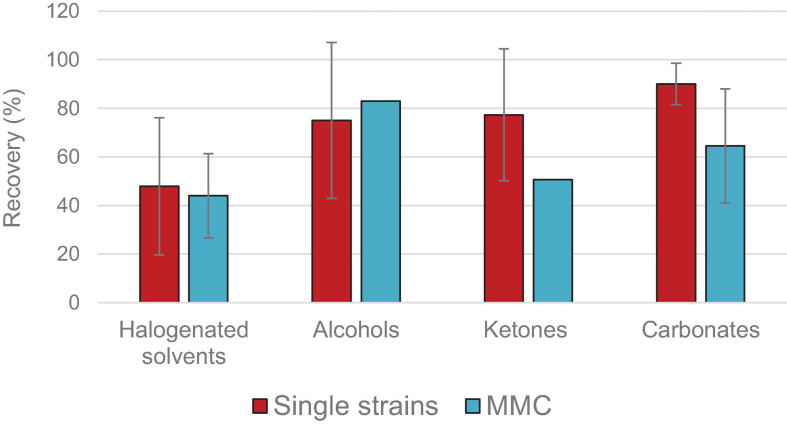
Mean recovery (%) of PHA extracted with solvents (halogenated solvents, alcohols, ketones, and carbonates) from single strains or MMC.

**FIGURE 5:**
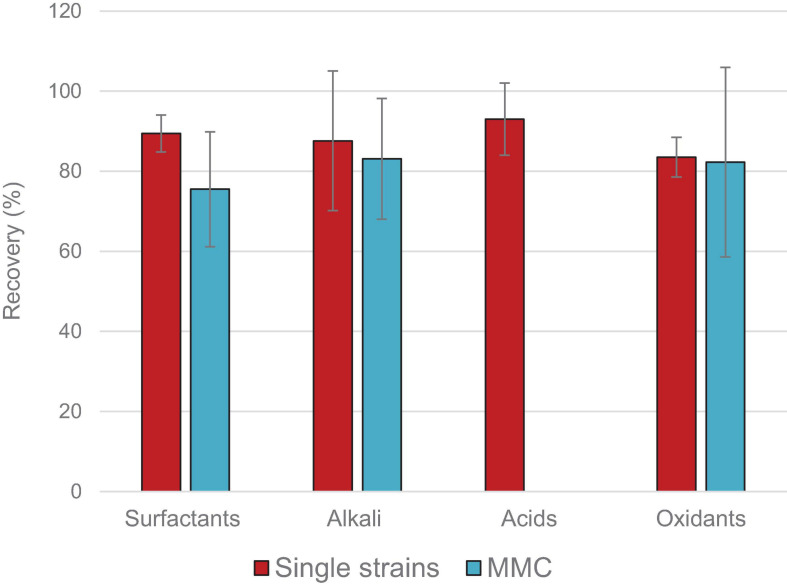
Mean recovery (%) of PHA through cellular lysis (with surfactants, alkali, acids, and oxidants) from single strains or MMC.

Mean PHB or PHA recoveries reported in the literature through solvent extraction or cellular lysis from single strains or MMC have been reported in Figures 4, 5.

The variability of results expressed here as standard deviations (SD) of all the values reported in the literature (Table 1) is very high, mainly due to the small amount of data related to solvent extraction on MMCs. For almost all solvent classes reported in the literature, PHA recovery is lower when MMC are extracted, confirming that the extraction process is more difficult for MMC than for single strain cultures. MMC are claimed to be more resistant to cell hydrolysis, probably because of a strong extracellular biomass matrix that contains the PHA accumulating cells. Furthermore, Patel et al. (2009) hypothesized a decrease in cellular fragility and therefore a minor amount of solvent able to access polymer granules because of a stronger non-PHA cellular materials (NPCM) and lower initial PHA content which results in a lower cell constrain.

The recovery data achieved from single strains are considerably higher (about 25%) when ketones or carbonates are applied; however, the literature does not report plenty of data about the extraction of MMC with ketones, therefore more data are needed for a more accurate analysis. The results obtained with halogenated solvents from single strains and mixed cultures are more homogenous and, in both cases, an average recovery below 50% is achieved. Alcohol extraction seems to be more efficient for MMC than for single strains (recovery of 83% vs. 75%), however, the result is not robust as there is just one study about the extraction of MMC with this kind of solvents that cannot be considered as a real benchmark (the same holds true for ketones applied to MMC, Werker et al., 2015).

The recovery of PHA from both single strains and MMC by applying a cellular lysis approach (Figure 5) is higher than what achievable by applying a solvent extraction; the average recoveries with surfactants, alkali, acids or oxidants are typically above 80%, whereas few solvents perform better than this threshold (Figure 4). The major differences between single strains and MMC are observed when cellular lysis is performed with surfactants (Figure 5), being the average recovery of PHA of 89 and 75%, respectively, whereas both the treatments with alkali and NaClO provide very similar results. By considering just the recovery percentage, surfactants, alkali, acids and oxidants perform well and similarly.

It is important to highlight that method that involve surfactants and alkali often include some pretreatments, especially with NaClO (Tables 2, 5) which has a strong oxidizing behavior capable of breaking cell membranes and enhancing the recovery.

### Molecular Weight

**FIGURE 6:**
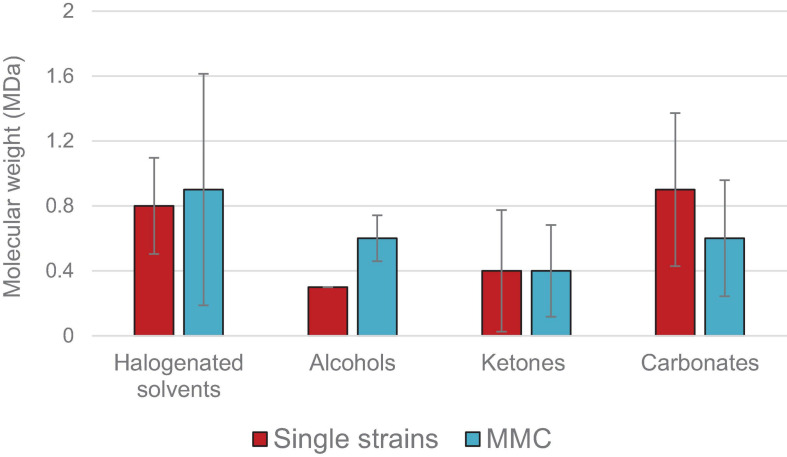
Mean molecular weight (MDa) of PHA extracted with solvents (halogenated solvents, alcohols, ketones, and carbonates) from single strains or MMC.

The molecular weights of polymers extracted with halogenated solvents are the highest among the ones achievable from both single strains and MMCs, ranging between 0.8 and 0.9 MDa, even if a large variability of the literature results is observed (Figure 6). Carbonates behave similarly, especially on single strains, and even in this case, a large variability is observed. Both carbonates and halogenated solvents seem to not shorten the length of polymer chains by side reactions (e.g., transesterification), and, at the same time, seem to be the best solvents for solubilizing high molecular weight PHA; this confirms alkyl carbonates as an effective and environmentally alternative for PHA extraction instead of other solvents.

In the literature there are few reports about the molecular weight of polymers extracted with ketones and alcohols, especially for MMCs, therefore the results are not very robust. The average PHA’s molecular weight extracted with ketones or alcohols from mixed and single strains is around 0.5 MDa but with high standard deviation in both cases (Figure 6); however, these lower values can be a confirmation of the suitability of ketones and alcohols for recovering “shorter” polymers than those extractable with carbonates and halogenated solvents.

**FIGURE 7:**
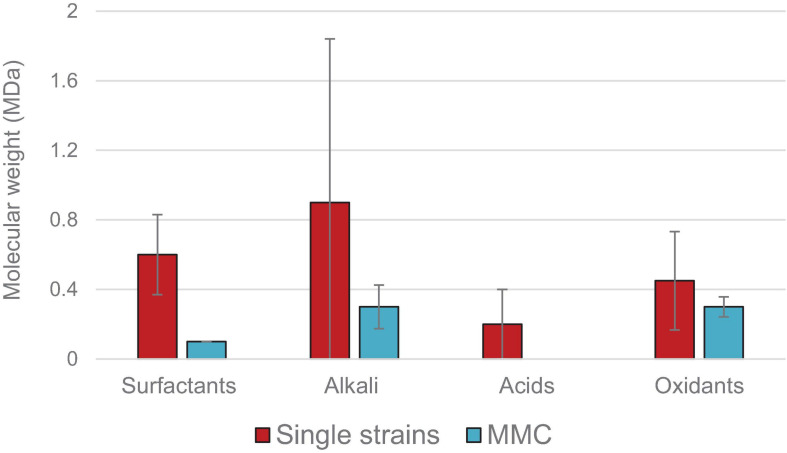
Mean molecular weight (MDa) of PHA extracted through cellular lysis (with surfactants, alkali, acids, and oxidants) from single strains or MMC.

The molecular weights of polymers extracted through cellular lysis (Figure 7) are largely variable. However, it seems quite clear that even if alkaline compounds do not compromise the length of PHA extracted from single strains (about 0.9 MDa), the same does not hold true for acids (0.2 MDa), oxidants (0.4 MDa) and surfactants (0.6 MDa). If the results got with NaClO are not surprising given its oxidizing nature, surfactants action is more unexpected; however, many data used here for creating the average molecular weight data achieved with surfactants come from a cellular lysis process composed by a pre-treatment with NaClO, followed by treatment with the surfactant itself. Another common pretreatment is a drying step prior to the polymer extraction (freeze-drying or thermal treatment); according to Koller et al. (2013), these pretreatments enhanced the polymer extraction causing the hydrolytic shortage of the polyester chains, more intrusive in the case of heat treatments than freeze-drying.

PHA recovered from MMC have much lower molecular weight averages than single strains, especially in the case of alkali and surfactants; this finding can be a confirmation of the higher resistance of MMC membranes than single strain membranes toward a “chemical” agent. Such resistance decreases when a strong oxidant is used, giving similar average molecular weights between single and mixed bacteria.

### Purity

**FIGURE 8:**
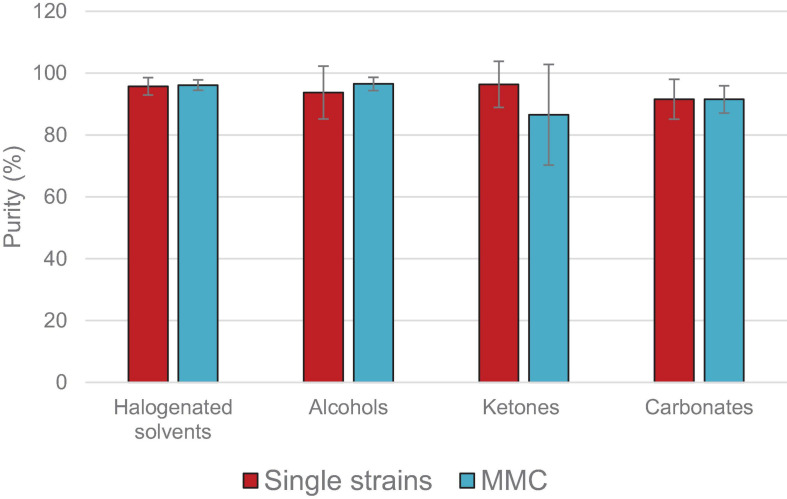
Mean PHA purity (%) achieved through extraction with solvents (halogenated solvents, alcohols, ketones, and carbonates) from single strains or MMC.

**FIGURE 9:**
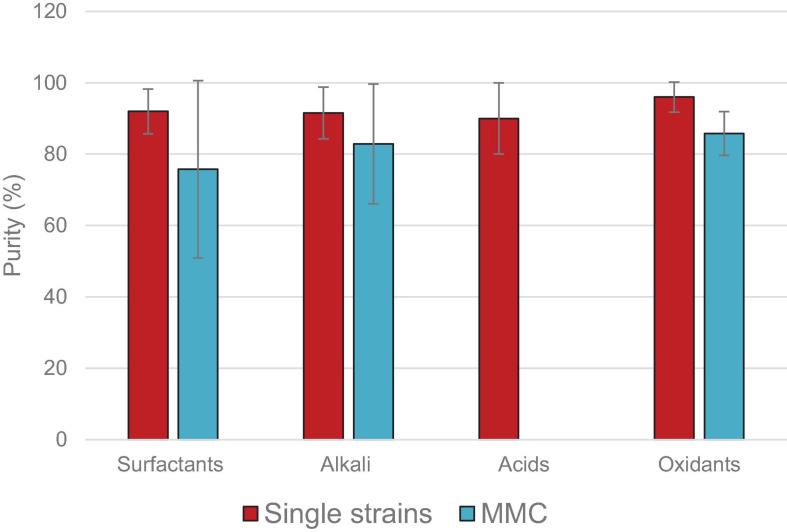
Mean PHA purity (%) achieved through cellular lysis (with surfactants, alkali, acids, and oxidants) from single strains or MMC.

The purity level of PHA extracted with solvents is very high (above 90%, Figure 8), independent by the type of solvent and the bacteria (single strains or MMC), the variability of this parameter is the lowest among those of all the parameters here used for comparing the extraction methods found in the literature (recovery and molecular weight). The PHA purity values obtained from cellular lysis approaches are lower, especially when MMCs are used (values below 90% with all the chemicals used, Figure 9). Larger variabilities can be noticed with this approach in comparison to solvent-based protocols, especially for less harsh treatments like the ones with surfactants and alkaline compounds.

## Conclusion

Considering the whole literature about PHA recovery from single strains and MMC it appears clear that a large variability of the results can be found. However, this variegate scenario strictly reflects the variability of bacteria, PHA initial content (that can influence the strength of the membranes), PHA type (homopolymers or co-polymers, and the ratio between short and long monomers), and recovery methods (temperature, pressure, need of pretreatments, concentrations of chemicals,…). Such variability is especially relevant for MMC, and this can be attributed to the variability of PHA content of MMC and the chemical features of MMC-derived PHA.

A focus on two of the most crucial parameters (purity and molecular weight) that can influence the applicability of the recovered PHA reveals that the solvent extraction approach generally gives purity above 98%, and molecular weight values above 0.8 MDa in the case of halogenated solvents and carbonates; lower values are got with ketones and alcohols, reasonably for a lower affinity of these class of solvents for PHA and not for a real shortening-chain effect.

The cellular lysis approach provides higher recoveries than the solvent-based approach, mainly due to the definition of “extraction yields” within such procedure. Given that the “extraction yields” are defined as the amount of PHA that stays in the solid phase, the PHA losses during cellular lysis are mainly due to severe PHA degradation. It follows that the two issues that affect the cellular lysis approach are the PHA purity and quality (e.g., molecular weight and PDI), and both fall in a quite wide range independent of single strains or MMC. The lower average purity obtained in some studies presented in the literature (70–80%) could be considered closer to a “refined microbial biomass” than “extracted PHA.”

## Author Contributions

GP developed the whole manuscript preparing and integrating the different parts. PG participated in the manuscript design and revised the manuscript. CS conceived the study and contributed to write the chemical aspects of the work and revised the manuscript. AZ contributed to collect and study the references cited. CT contributed to write the aspects of efficiency issues of PHA extraction and revised the manuscript. All the authors read and approved the final manuscript.

## Conflict of Interest

The authors declare that the research was conducted in the absence of any commercial or financial relationships that could be construed as a potential conflict of interest.
